# Salt or Cocrystal Puzzle Solved by Mechanochemistry: The Role of Solvent in the Pamoic Acid Case Study

**DOI:** 10.1002/chem.202500956

**Published:** 2025-05-19

**Authors:** Andrea Daolio, Michele Prencipe, Temitope Abodunrin, Paolo Pelagatti, Paolo Pio Mazzeo, Alessia Bacchi

**Affiliations:** ^1^ Dipartimento di Scienze Chimiche, della Vita e della Sostenibilità Ambientale Università degli Studi di Parma Viale delle Scienze, 17A Parma 43124 Italy; ^2^ CSGI: Center for Colloid and Surface Science Via della Lastruccia 3 Sesto Fiorentino (FI) 50019 Italy; ^3^ Department of Physical Sciences Landmark University, P.M.B Omu Aran 1001 Nigeria; ^4^ Centro Interuniversitario di Reattività Chimica e Catalisi (CIRCC) Via Celso Ulpiani 27 Bari 70126 Italy; ^5^ Biopharmanet‐TEC University of Parma Parco Area Delle Scienze 27/A Parma 43124 Italy

**Keywords:** cocrystal, crystallography, LAG, mechanochemistry, packing

## Abstract

Many drugs are nowadays marketed as salts. Cocrystallization is also emerging as a convenient tool to modify in vivo activity of pharmacologically active compounds. Given the marked difference in physicochemical properties between salts and cocrystals, the possibility of obtaining crystalline systems composed of the same building blocks in their neutral or charged form is desirable. Pamoic acid (PAM) is widely used in pharmaceutical formulation as pamoate salt, and we here propose a unique synthetic strategy to obtain a cocrystal of **PAM** rather than a salt by tuning mechanochemical conditions. Our findings have been corroborated by means of computational analyses, relating to the noncovalent interactions in the crystal structure with the formation of the different crystalline forms. A detailed analysis of the structure containing **PAM** in its neutral, anionic or dianionic form present in the Cambridge Structural Database (CSD) helped generalizing our results.

## Graphical abstract

Pamoic acid:quinoline cocrystal and pamoate:quinolinium salts can be synthetized by mechanochemistry tuning the reaction conditions. Computational analysis of the crystal packing reveals the main factors driving the formation of either forms. This approach and subsequent rationalization pave the way to the synthesis of pharmaceutically active multicomponent systems with tunable properties.

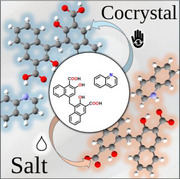

## 1 Introduction

In vivo activity of active pharmaceutical ingredients (APIs) is often determined by their solubility, lipophilicity, and release profile, in turn dictated by the phase in which they are administered.^[^ ^1^ ^]^ Design of multicomponent crystalline phases, either salts or cocrystals, involving the API and one or more molecular partner (i.e., coformers) is one of the most efficient strategies to tune such properties.^[^ ^2^ ^]^

A salt and a cocrystal represent two extremes of a continuum of supramolecular entities in which the inclusion of the API is brought by the proton transfer or by noncovalent interactions (e.g., hydrogen bonds or π‐π stacking), respectively.^[^ ^3^, ^4^, ^5^ ^]^ Salification of APIs is a general practice to improve the aqueous solubility of drugs. To date, more than 50% of drugs are marketed as salts.^[^ ^6^ ^]^ In recent years, cocrystallization has also emerged as an efficient practice to alter the physicochemical properties of APIs.^[^ ^7^, ^8^, ^9^, ^10^ ^]^ Moreover, the latter approach is not limited to ionizable drugs. The accurate choice of the coformer can increase the melting point of volatile APIs,^[^ ^11^ ^]^ alter the water solubility of drugs,^[^ ^12^ ^]^ and modify their release kinetic.^[^ ^13^ ^]^ Given the possibility of exploiting such properties, the study of the crystalline arrangement of coformers routinely employed in those formulations is timely.^[^ ^14^, ^15^ ^]^ A detailed understanding of the chemical, energetical, and conformational properties of a coformer would guide the design of specific formulations to be used as pharmaceuticals, possibly moving from heuristic principles to accurate predictions.^[^ ^16^, ^17^ ^]^ In parallel, the ability to produce such materials in bulk with virtually no waste would be incredibly beneficial for the field.

Mechanochemistry is gaining traction as a cost‐effective and sustainable route to obtain such compounds.^[^ ^18^, ^19^, ^20^, ^21^ ^]^ Indeed, different classes of organic,^[^ ^22^ ^]^ inorganic,^[^ ^23^ ^]^ and metal‐organic^[^ ^24^ ^]^ materials are obtained following this approach. For its wide range of applications and overall sustainability in 2019 IUPAC listed mechanochemistry as one of the 10 chemical innovations that will change our world.^[^ ^25^ ^]^ While many traditional solution‐based chemical reactions can be carried out mechanochemically, through the latter is often possible to promote the formation of novel phases and adducts not accessible via more conventional “wet” techniques.

**Figure 1:**
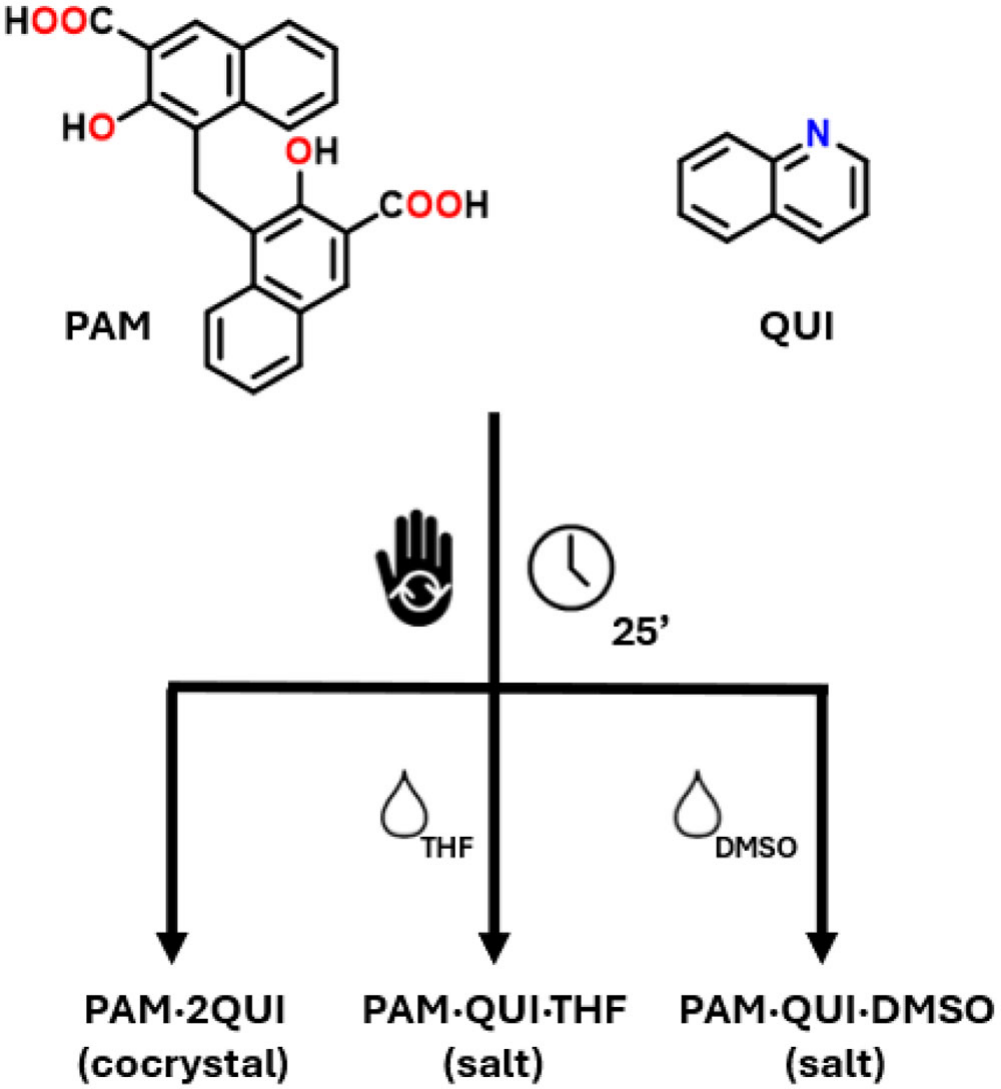
Scheme of pamoic acid (PAM), quinoline (QUI), and summary of the grinding conditions employed to obtain all the crystal structures analyzed in this study.

Pamoic acid (4,4′‐Methylenebis(3‐hydroxynaphthalene‐2‐carboxylic acid), **PAM** in Figure 1, p*K*a 2.67)^[^ ^26^ ^]^ is an inexpensive, nontoxic widely employed excipient in drug formulations.^[^ ^27^ ^]^ This compound presents many different functional groups capable of attractively interacting with other molecules while maintaining relative conformational freedom due to the methylene group linking two aromatic cores. Pamoate salts are incredibly effective in altering the solubility of a wide range of bioactive drugs.^[^ ^28^, ^29^ ^]^ While a comprehensive list of said formulations is ever‐expanding and exceeds the scope of this article, we cite pyrantel pamoate,^[^ ^30^ ^]^ oxantel pamoate,^[^ ^31^ ^]^ and olanzapine pamoate hydrate^[^ ^32^ ^]^ as relevant examples. They all contain nitrogen atoms capable of accepting the proton from one of the two carboxylic acids of **PAM**, which supported our choice to study the crystal habits of the excipient employing quinoline as a model compound (**QUI** in the following, p*K*a 4.94).^[^ ^33^ ^]^

The vast majority of drug formulations containing **PAM** are salts in which the excipient is present as a mono‐ or di‐anion. To the best of our knowledge, no cocrystal containing **PAM** is sold. This is also reflected in the abundance of **PAM** salts with respect to cocrystals in the Cambridge Structural Database (CSD)^[^ ^34^ ^]^ in which, excluding DMF and NMP solvates, a single cocrystal of **PAM** is reported.

Several in‐depth studies have been performed on the crystal arrangement of pamoate salts. The structural features of many adducts have been systematically characterized, inferring that the crystalline arrangements seem to be mainly dictated by the charge of **PAM**, regardless of the cation employed in the formulation of pamoate salts. Referring specifically to the works of Haynes et al.,^[^ ^35^, ^36^, ^37^, ^38^, ^39^, ^40^ ^]^ two main forms of pamoate crystals have been studied. In form A, by far the most common, **PAM** is present in its monoanionic form, while in form B it is present as a dianion. The role of the solvent in the formation of a specific form of pamoate salts has also been tackled.^[^ ^41^, ^42^, ^43^ ^]^ Cocrystals in which **PAM** is present in its neutral dicarboxylic acid form were rarely found even when weak bases were present. A reliable way to obtain **PAM** cocrystals as opposed to pamoate salts would expand the range of possible drug formulations to be designed, not limited anymore to ionizable drugs.

In this work, by rationally tuning mechanochemical conditions in which the cocrystallization was carried out, we were able to obtain three novel crystalline phases; two can be described as pamoate salts of quinolinium (form A), while one is a cocrystal in which **PAM** is present as its neutral carboxylic acid. We hereby propose to designate this peculiar crystalline packing as form C.

Given the remarkable difference salts and cocrystals show in terms of solubility^[^ ^44^ ^]^ and the ubiquity of **PAM** in drug formulations, we deemed this system worth a more in‐depth study, especially in providing tentative guidelines for obtaining one form or another. The two compounds were computationally analyzed in terms of their molecular electrostatic potential (MEP) and the most relevant interactions found in the crystals were analyzed through quantum theory of atom in molecules (QTAIM), Noncovalent Interaction Index (NCI), and energy decomposition analysis (EDA, at the SAPT0 level), providing valuable insights into the relative stabilities of these forms. At last, ranking the stability of the forms in terms of lattice energy provided useful insights into the formation of one form over another.

A detailed analysis of the **PAM** crystal structures available on the CSD has been performed, helping to contextualize the novelty of our results, and establishing the main interaction patterns that **PAM** or pamoate salts can engage into.

## 2 Experimental Section

### 2.1 General

All reagents were purchased from Sigma Aldrich and used without further purification.

### 2.2 Synthesis of PAM·QUI·THF

39 mg of **PAM** (0.1 mmol, 1 equivalent) and 12.5 µL of **QUI** (0.1 mmol, 1 equivalent) and 17.6 µL of THF (0.22 mmol, 1 equivalent plus Liquid Assisted Grinding (LAG), *η* = 0.2) were manually ground for 25 minutes at room temperature (25 °C) in an agate mortar. Single crystals of PAM·QUI·THF were obtained by dissolving 39 mg of **PAM** and 12.5 µL of **QUI** in approx. 5 mL of THF in a clear borosilicate vial, letting the solvent slowly evaporate. Prismatic, yellowish crystals suitable for single crystal X‐Ray diffraction were obtained at the bottom of the vial after 48 hours.

### 2.3 Synthesis of PAM·QUI·DMSO

39 mg of **PAM** (0.1 mmol, 1 equivalent), 12.5 µL of **QUI** (0.1 mmol, 1 equivalent), and 19.6 µL of DMSO (0.28 mmol, 1 equivalent plus LAG, *η* = 0.2) were manually ground for 25 minutes at room temperature (25 °C) in an agate mortar. Single crystals of PAM·QUI·DMSO were obtained by dissolving 39 mg of **PAM** and 12.5 µL of **QUI** in approx. 5 mL of DMSO in a clear borosilicate vial, letting the solvent slowly evaporate. Prismatic, yellowish crystals suitable for single crystal X‐Ray diffraction were obtained at the bottom of the vial after 48 hours.

### 2.4 Synthesis of PAM·2QUI

39 mg of **PAM** (0.1 mmol, 1 equivalent) and 25 µL of **QUI** (0.2 mmol, 2 equivalents) were manually ground for 25 minutes at room temperature (25 °C) in an agate mortar. While grinding, the viscous paste produced gradually evolved into a fine yellowish powder. Single crystals of PAM·2QUI were obtained by dissolving 39 mg of **PAM** and 12.5 µL of **QUI** in approx. 5 mL of DMSO in a clear, borosilicate vial, letting it rest in a chamber containing approx. 10 mL of EtOH. Small, poorly diffracting plate‐like yellowish crystals were collected at the bottom of the vial after 24 hours.

### 2.5 Diffraction experiments

PXRD data of the crystals were collected on a Rigaku Smartlab XE diffractometer in θ–θ Bragg–Brentano geometry with Cu Kα radiation. The samples were placed on glass supports and exposed to radiation (5° ≤ 2θ ≤ 30°) at a scan rate of 10°/minute. The diffracted beam was collected on a 2D Hypix 3000 solid‐state detector; 5° radiant Soller slits were used as a compromise for high flux and moderate peak asymmetry at low angles. A beam knife and antiscatterer air component were used to mitigate the scattering at a low 2θ angle.

Crystal structures were obtained by collecting the diffracted intensities with a CMOS Photon II 2D detector on a Bruker D8 Venture diffractometer, equipped with a kappa goniometer and an Oxford Cryostream. Data collection was performed either with a microfocused Cu *K*α radiation (*λ* 1.54 178 Å) or microfocused Mo Kα radiation (*λ* = 0.71 073 Å). Measurements were performed at 150 K for PAM·QUI·THF and 300 K for PAM·QUI·THF and PAM·2QUI. Lorentz polarization and absorption corrections were applied for all the experiments. Data reduction was carried out using APEX v4 software.^[^ ^45^ ^]^ The structures were solved by direct methods using SHELXT^[^ ^46^ ^]^ and refined by full‐matrix least‐squares on F2 with anisotropic displacement parameters for the non‐H atoms using SHELXL2016/6.^[^ ^47^ ^]^ Absorption correction was performed on the basis of a multiscan procedure using SADABS. Structural analysis was aided by use of the program PLATON.^[^ ^48^ ^]^ The hydrogen atoms were calculated in ideal positions with isotropic displacement parameters set to 1.2 Ueq of the attached atom. Crystal data are reported in Table S2 (Supporting Information).

### 2.6 Computational details

For the analysis of the MEP structures of **QUI**, **PAM**, quinolinium cation, and pamoate anion were fully optimized in vacuum with the quantum chemistry package Gaussian16.^[^ ^49^ ^]^ In a different set of experiments, namely for the analysis of noncovalent interactions as present in the crystalline adducts (QTAIM, NCI, and Symmetry‐Adapted Perturbation Theory (SAPT) analyses), relevant dimers were extracted from the crystal structures and optimized in those molecular geometries (optimizing just the hydrogen atom positions while freezing the others). This approach is routinely used in the analysis of noncovalent interactions.^[^ ^50^, ^51^ ^]^ Both sets of optimizations were performed at the at the M06‐2x/cc‐PVTZ level of theory on a “Ultrafine” grid with default cutoffs for forces and step size. MEP surfaces have been computed using the M06‐2x/cc‐PVTZ level of theory of the fully optimized (isolated) molecules and, according to Bader's suggestion, represented using 0.001 a.u. isosurface value of electron density to map the electrostatic potential as customary for those simulations.^[^ ^52^, ^53^ ^]^ QTAIM and NCI analysis was performed using the AIMAll^[^ ^54^ ^]^ software on the dimers extracted from the crystal structures and optimized freezing all nonhydrogen atoms. SAPT analysis was performed on the dimers extracted from the crystal structures and optimized freezing all nonhydrogen atoms by means of the Psi4 software.^[^ ^55^ ^]^ The analysis was performed at the SAPT0 level using the jun‐cc‐pVDZ basis set.^[^ ^56^ ^]^ Lattice energies were calculated using CrystalExplorer21^[^ ^57^ ^]^ at CE‐B3LYP level of theory, considering for each molecule all of the other surrounding molecular entities in a sphere of radius 30 Å. The pair interaction energies were averaged according to the number of independent molecules in the asymmetric unit (Z’) for lattice energy calculations.

### 2.7 Statistical survey

The structures available on the CSD were retrieved with the aid of the software conquest, part of the CCSD suite,^[^ ^34^ ^]^ and divided into subgroups owning to the nature of **PAM** moiety (neutral, anion, or dianion). Relevant geometrical parameters were extracted from the hitlists either by the “ADD3D” option available on the software or by hand. A careful inspection of the structures ensured that no error was present.

## 3 Results and Discussion

**Figure 2:**
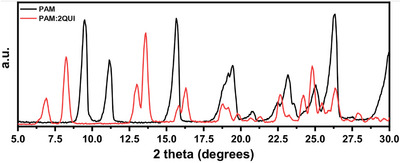
Comparison of the PXRD of pamoic acid and PAM·2QUI in a 2ϴ range of 5–30°. Pamoic acid (PAM) is reported in black, PAM·2QUI is reported in red.

Grinding **PAM** and **QUI** in the conditions described in the experimental sections produces crystalline phases clearly different from that of pamoic acid, as confirmed by PXRD analysis in a 2ϴ range of 5–30° (Figure 2). Our attempts to obtain crystals suitable for single crystal X‐Ray diffraction brought to three different structures displaying a calculated PXRD consistent with those obtained by grinding experiments. The products were prepared by neat/LAG grinding, and by precipitation. Products with 1:1 and 1:2 stoichiometry were obtained corresponding to the initial ratio of the components; the products however differed also in the ionic/neutral nature of the components, depending on the presence of solvent in the synthetic procedure. The structures will be discussed in the following.

**Figure 3:**
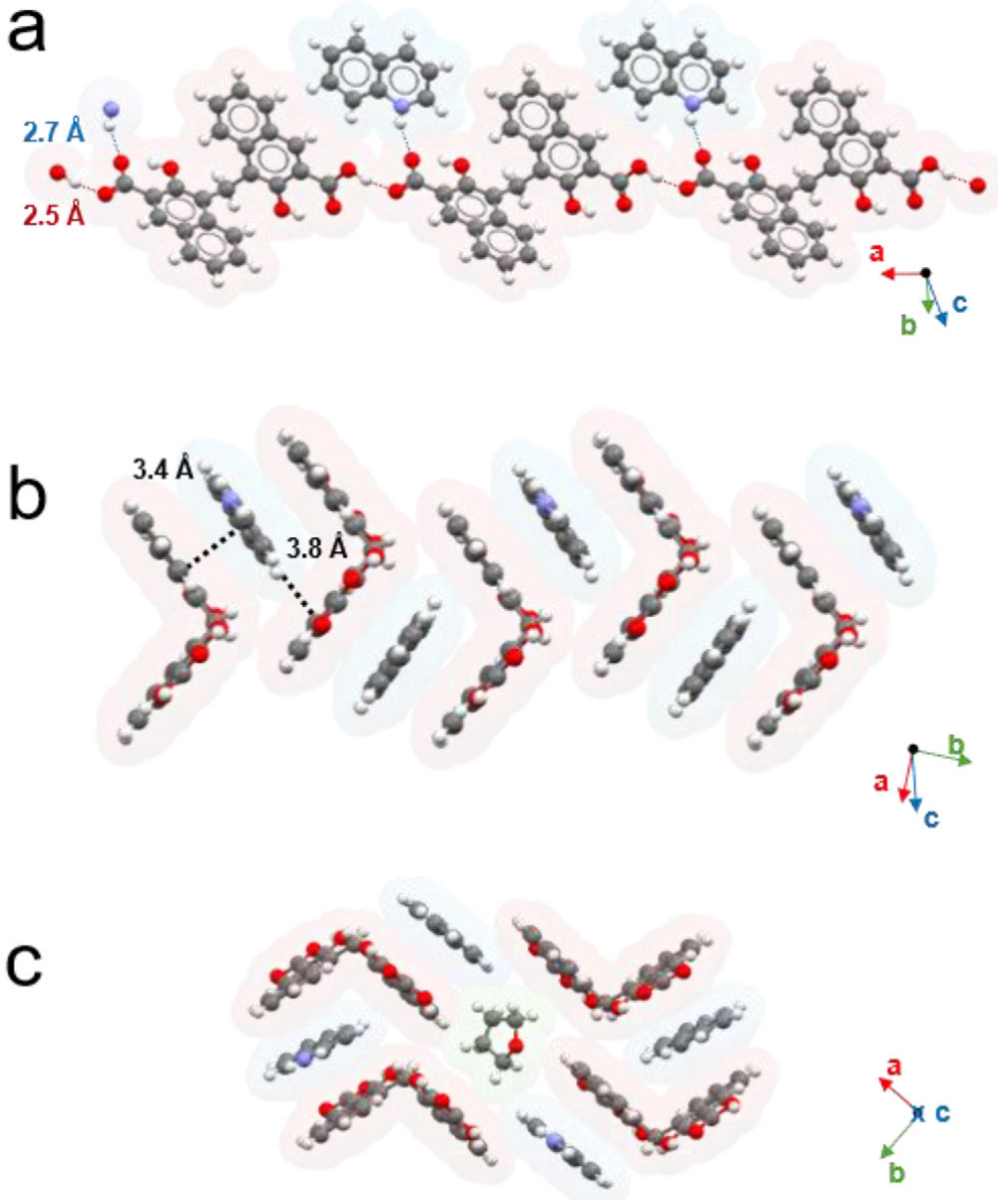
Molecular assembly of PAM·QUI·THF. Each molecule is superimposed to its van der Waals surface with arbitrary colors; **PAM** units in red, QUI units in light blue, and solvated molecules (THF) in green. a) Details of the hydrogen bond network. Red dotted lines represent hydrogen bonds between **PAM** units and light blue dotted lines hydrogen bonds between **PAM** and **QUI** units. O···O and O···N distances are highlighted. b) Details of the π‐ π stacking network. Average distances between **QUI** and different units of **PAM** are highlighted. c) Details of the surroundings of one solvated THF molecule.

The isostructural PAM·QUI·THF and PAM·QUI·DMSO crystallize in the monoclinic space group *P 21/c* with a Z of 4. According to the extensive similarities between the two networks (Table S2, Supporting Information) they will be described together. In both structures **PAM** is present in its monoanionic form, engaging in strong hydrogen bonds between the carboxylic acid of a molecular entity and the carboxylate of another, extending approximately along the *a‐*axis by forming chains (Figure 3A). The second oxygen atom of the carboxylate group is additionally engaging in a second hydrogen bond with the quinolinium cation, which sits approximately perpendicular to the “anionic” subunit of **PAM** and parallel to the “neutral” one, engaging in short π‐π stacking interactions with different **PAM** units (the mean distance between the centroid of **PAM** and the two planes described by the pamoate subunits are 3.41 and 3.40 Å for PAM·QUI·THF and PAM·QUI·DMSO, respectively, Figure 3B), linking together different carboxylate chains, and forming the 3D network. Both hydroxyl groups of pamoate salt engage in S(6) hydrogen bond motives^[^ ^58^ ^]^ with the closest carboxylic oxygen, so that the carboxylate of **PAM** is engaging in three different hydrogen bonds, and the carboxylic acid in just one (Figure 3A). As visible from Figure 3C this peculiar arrangement produces cavities that are filled with solvent molecules (THF for PAM·QUI·THF and DMSO for PAM·QUI·DMSO), which additionally establish dispersive interactions with their surroundings. The distance between the carboxylate oxygen and the quinolinium nitrogen is 2.72(1) Å for PAM·QUI·THF and 2.78(1) Å for PAM·QUI·DMSO and that of the two oxygens involved in the chain formation is 2.48(2) Å for PAM·QUI·THF and 2.47(1) Å for PAM·QUI·DMSO, indicating in both cases quite strong hydrogen bonds. The geometrical characteristics of the two salts are consistent with those owning to form A as described by Haynes.^[^ ^43^ ^]^

**Figure 4:**
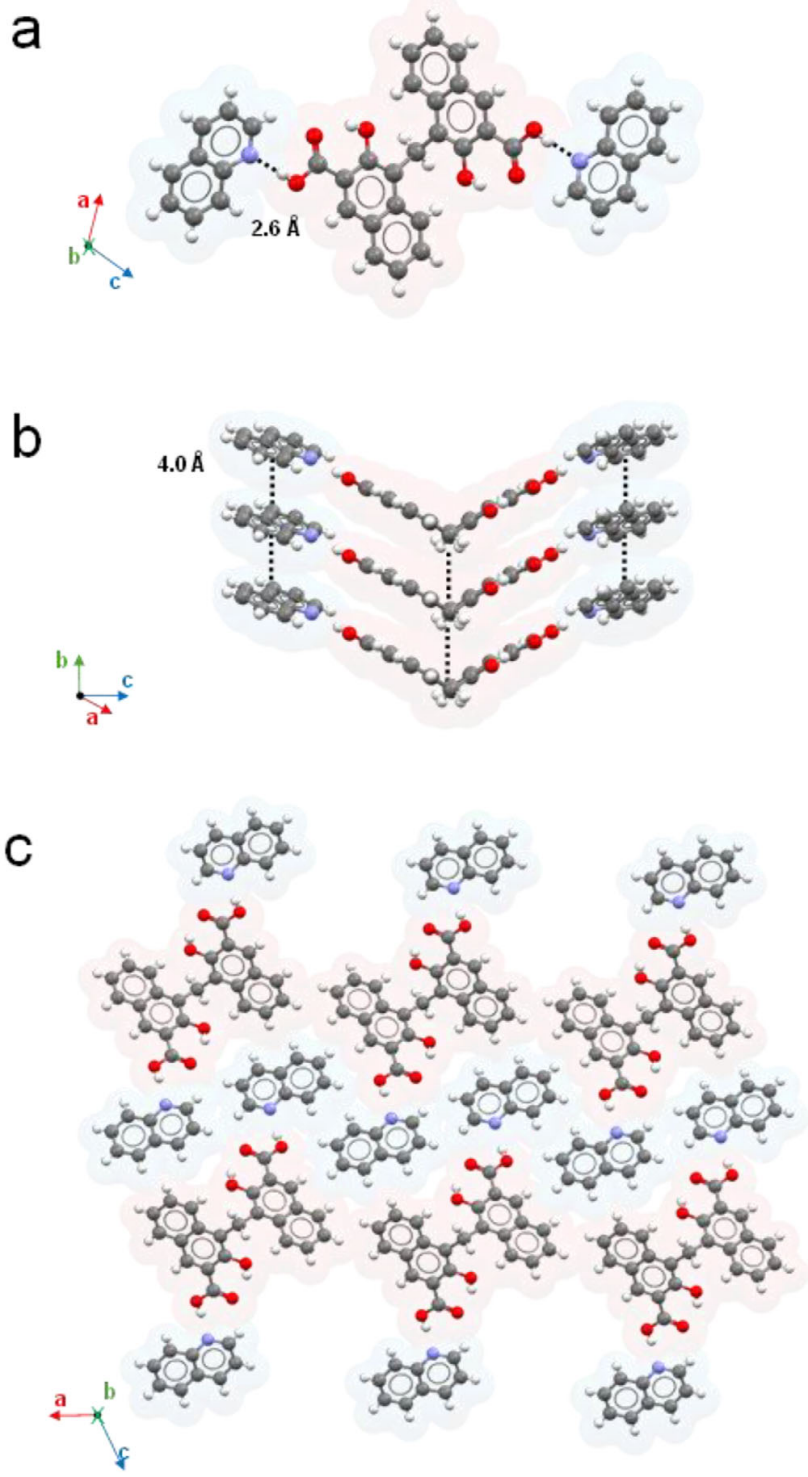
Molecular assembly of PAM·2QUI. Each molecule is superimposed to its van der Waals surface with arbitrary colors, **PAM** units in red, **QUI** units in light blue. a) Trimeric assembly of one unit of **PAM** and two units of QUI. Hydrogen bonds between **PAM** and **QUI** units are black dotted lines. O···N distances are highlighted. b) Details of the π‐π stacking network. Average distances between **QUI** and different units of **PAM** are highlighted. c) Molecular assembly of the columnar, trimeric entities, as viewed from the *b*‐axis of the crystal.

**PAM·2QUI** instead crystallizes in the monoclinic space group *C 2/c* and exhibits a different assembly from the first two structures, with **PAM** molecules present in neutral form (Figure 4). As further confirmation, the measured lengths of the C─O and C = O covalent bonds are consistent with those of a carboxylic acid (1.32(2) and 1.23(1) Å, respectively). The two carboxylic acids are involved in hydrogen bonds with **QUI** units, which this time sit parallel to the aromatic portion of the **PAM** unit they are interacting with (approximately along the *c‐*axis of the structure). The O···N distance in this case is slightly shorter than the other two structures, measuring 2.64 Å. The intramolecular hydrogen bond between the hydroxyl group and the carboxylic oxygen is preserved. The 3D network is then achieved by means of different π‐π stacking interactions in which both **PAM** and **QUI** stack into columns along the *b‐*axis of the crystal (4.02 Å). This particular packing is almost unprecedented, and we propose to designate it as form C.

The Δp*K*a rule is routinely employed when trying to discriminate the formation of a cocrystal or a salt given an acid/base pair.^[^ ^59^ ^]^ Δp*K*a of the two compounds is 2.27 which, even if skewed toward the formation of salts, is in the region accommodating what has been referred to as the salt/cocrystal continuum,^[^ ^60^ ^]^ consistent with the formation of either cocrystals or salts. Namely, this small difference is not sufficient to discriminate the formation of either a salt or a cocrystal and has been proven statistically insignificant for predictive purposes^[^ ^61^ ^]^ moreover, the relative stabilities of the crystalline phases is expected to play an additional role which is not covered by this simple analysis.

**Figure 5:**
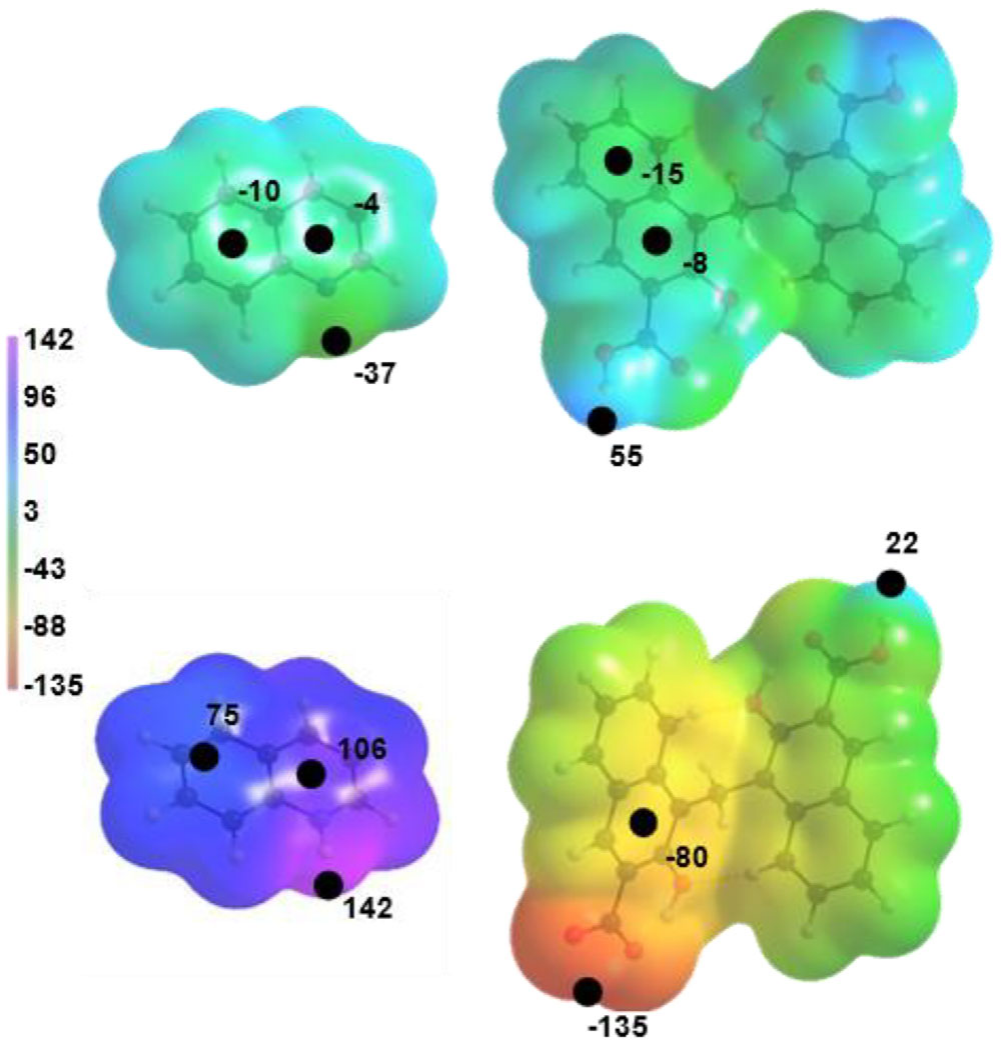
Molecular electrostatic potential (MEP) mapped onto the 0.01 a.u. electron density isosurface of **PAM** and **QUI** in their neutral (top) and charged (bottom) forms. Scale of the MEP is the same for all four compounds. Relevant critical points are highlighted as black circles. Values of their MEP are reported near the circles. All values are in kcal/mol.

Further analyses, focusing on the interaction energies as present in the crystal structures, were performed. Our investigation into the crystal packing of these two compounds started by computing the MEP of **PAM** and **QUI** both in their neutral and charged forms. Figure 5 shows the MEP surfaces of the four molecules studied herein in the same energetic scale. When **PAM** and **QUI** are neutral (as in PAM·2QUI) the maximum and minimum of their MEP is located on the carboxylic acid hydrogens of **PAM** (55.8 kcal/mol) and on the nitrogen of the pyridyl moiety of **QUI** (−37.1 kcal/mol), respectively. The protonated form of **QUI** and the anionic **PAM** show higher values according to the formal charge of the entire molecule (141.8 kcal/mol and −135.0 kcal/mol, respectively). The quinolinium cation electrostatic potential over the ring plane ranges from 100 to 144 kcal/mol, while for the neutral **QUI** the maximum on the same area is just 12 kcal/mol. For **PAM** and pamoate anion, the most negative region over the aromatic portion accounts for −8.2 and −56.5 kcal/mol, respectively. Being interested in how the crystal packing and the mechanochemical conditions influence the formation of either one of the two forms, additional calculations were performed to characterize the arrangements also in energetical terms. Namely, we performed QTAIM analysis, NCI, and EDA on selected dimers, focusing on the first neighbors of both **PAM** and **QUI**, to assess the strength of the hydrogen bonds and of π‐π stacking between them.

**Figure 6:**
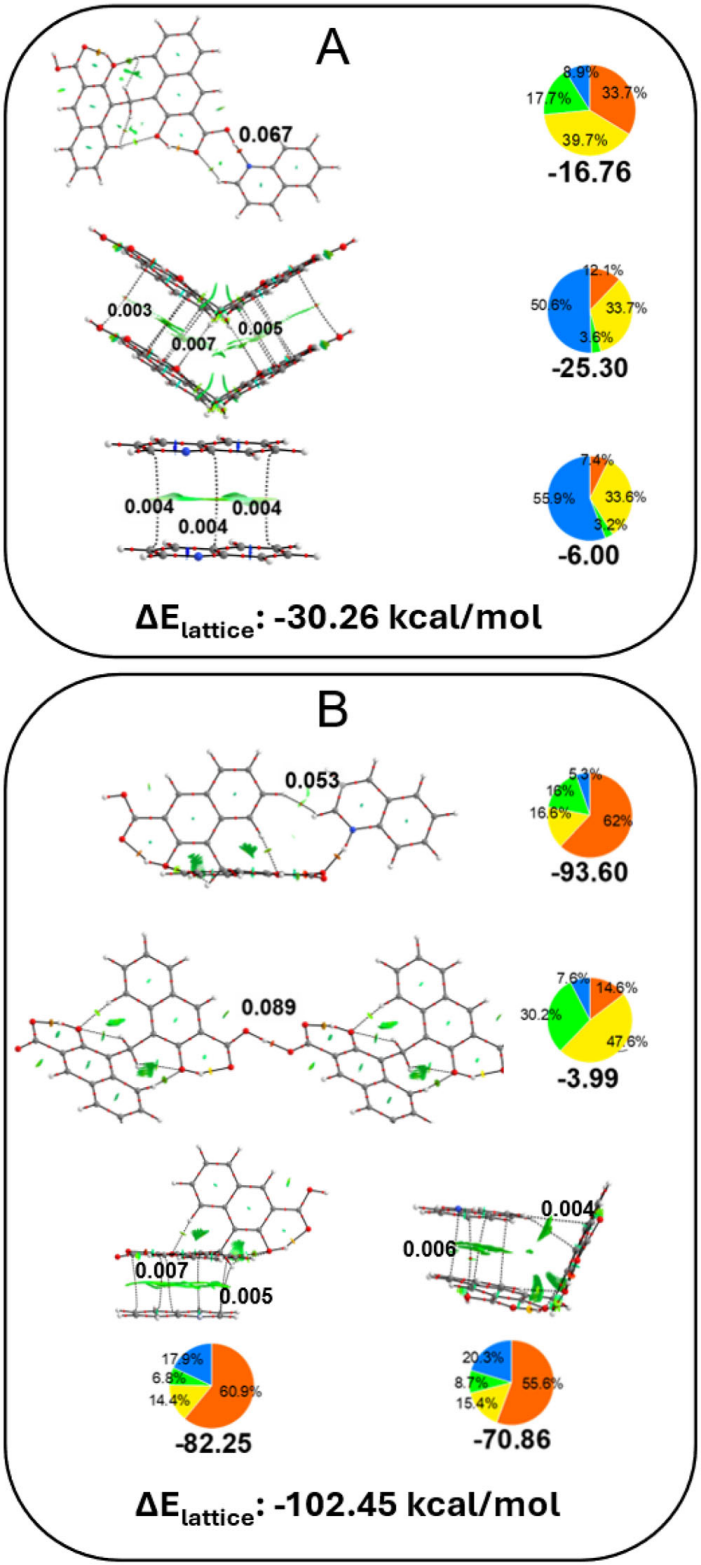
Details of QTAIM and SAPT0 analyses performed on (A) PAM·2QUI and (B) PAM·QUI·THF. BCPs are green dots spheres and bond paths are black lines. The values of electron density (*ρ*) at relevant BCPs are listed near the spheres as e−/bohr3. Values of the SAPT0 interaction energies are listed near the dimer, and the energy decomposition is given as a histogram. Orange coulombic, green induction, yellow exchange, blue dispersion. All values of SAPT are given in kcal/mol. At the bottom of each frame, the lattice energy estimate with CrystalExplorer is highlighted.

A summary of the calculations can be found in Figure 6. QTAIM analysis is able to outcome the bond critical points (BCPs) linking the molecules considered. In the case of PAM·2QUI, QTAIM finds a value of 0.067 e^−^/bohr^3^ between the carboxylic hydrogen of **PAM** and the nitrogen of **QUI**, aided by an additional weaker hydrogen bond established by a carbonyl oxygen and another hydrogen of **QUI**. Meanwhile, the electron density at the BCPs in the π‐ π stacked dimers is remarkably weaker, which is common if the dimer is mostly stabilized by dispersive forces. A similar situation is found in PAM·QUI·THF, where the electron density related to the formation of the hydrogen bonds between **PAM** and **QUI** is comparable to the one in PAM·2QUI.

EDA further complements the interaction analysis by deconvoluting the total interaction energy into different contributions. As visible from the simulations, it can be stated that the hydrogen bond interactions are dominated by electrostatic and polarization contributions. While dispersive forces still play a role in the stabilization of the dimers, a major electrostatic contribution, undoubtedly arising from the interaction of charged species, is also present, and accounts for E*i*_n_ three to four times stronger than those present between the neutral counterparts. Lastly, even if established between anions and likely stabilized by the other interactions in the solid state, the hydrogen bond between two **PAM** units in PAM·QUI·THF is still stabilizing thanks to a major polarization contribution.Lastly, we computed the lattice energy of the two forms through the software CrystalExplorer (Figure 6). As expected, PAM·QUI·THF is the most stabilized framework, while in PAM·2QUI the stabilization is lower.

Overall, a few indications can be drawn from the analyses. It can be inferred that salts of form A are more stable than cocrystals of form C. In the former, the most stabilizing forces arise from the strong electrostatic contribution induced by the presence of charges between **PAM** and **QUI** units. In the latter, hydrogen bonds link **PAM** and **QUI**, which in turn assemble through dispersive interactions between molecules of the same type. Consideration must be given to the solvent molecules in PAM·QUI·THF. As visible in Figure 2C solvent molecules sit between different **PAM** and **QUI** units, preventing repulsive interactions between charges of the same sign. On the other hand, an arrangement similar to form C cocrystals would be impossible for charged species due to electrostatic repulsive interactions. It can be inferred that the presence of a suitable solvent provides the necessary separation between like charges to allow for the formation of a salt of type A, while stabilizing the packing through dispersive forces (Table S.15, Supporting Information). In its absence, **PAM** and **QUI** can only proceed to the formation of a cocrystal of form C.

An in‐depth survey of the structures available on the CSD in which **PAM** is present, either in its neutral or anionic forms, is especially helpful in contextualizing our results. As of November 2024, on the database are present 94 structures containing **PAM** (structures including metal complexes or those where **PAM** is used as Metal Organic Frameworks ligands were omitted). Only five show **PAM** in its neutral form. Of them, three are either **PAM** or **PAM** solvates, and only one structure (**PAM**·lutidine 1:2, refcode TAPFIB^[^ ^42^ ^]^) crystallizes with a packing reminiscent of PAM·2QUI. The remnant structures can be divided into form A salts (54 hits) or form B salts (22 hits). In most of the form A salts, a hydrogen bond between two subunits of **PAM** is present, and when absent is often substituted by a hydrogen bond between two **PAM** units and a solvated water molecule. In this form, short π‐π stacking between **PAM** and the cation is present. When the chemical nature of the cation precludes the formation of this interaction (i.e., when the cation does not possess any aromatic moiety) it is usually substituted by quite short CH···π interactions between the two. The data presented also help in rationalizing the high number of solvated salts of form A and B present in the CSD (about 80% of the total). In contrast, the two cocrystals in which **PAM** is present as a dicarboxylic acid still displays hydrogen bonds between **PAM** and weak bases, but the π‐π stacking is preferably established between different units of **PAM** than with its coformer. The data presented herein are in complete accordance with those owning to the structures we obtained in this study and, although a rigorous computational analysis of all the structures available exceeds the scope of this article, strongly suggest that our finding of the PAM‐QUI system may have a more general significance.

## 4 Conclusion

We hereby presented a case study whereby the choice of mechanochemical conditions is crucial for driving the formation of a specific multicomponent crystalline form. PAM, one of the most used excipients, was employed as a coformer in conjunction with a model compound returning salts or neutral cocrystals depending on the mechanochemical conditions in which the crystallization was carried out. While still in its infancy, mechanochemistry is undoubtedly a much greener route to produce cocrystals as opposed to more traditional solution methods. The possibility of obtaining **PAM**/pamoate multicomponent crystalline phases in quantitative yield with virtually no waste might be strongly beneficial for large‐scale medicinal chemistry applications. Additionally, in this paper we demonstrated a possible route to obtain **PAM** cocrystals in place of the much more common pamoate salts. Since the release profile of neutral (cocrystals) or charged (salts) adducts are often dramatically different, this work might help in the formulation of the more efficient form for pharmaceutical applications.

## Supporting Information

The Supporing Information comprise Thermal analyses, X‐ray Powder diffraction refinements, crystallographic and computational details. Single crystal X‐ray structures have been deposited on the CSD under deposition numbers 2429995–2429997.

## Conflict of Interests

The authors declare no conflict of interest.

## Supporting information



Supporting Information

## Data Availability

The data that support the findings of this study are available in the supplementary material of this article.
