# Supplementary material for: Salt or Cocrystal Puzzle Solved by Mechanochemistry: The Role of Solvent in the Pamoic Acid Case Study
Source: Chemistry. 2025 May 19;31(33):e202500956. doi: 10.1002/chem.202500956 (PMC12160972; doi:10.1002/chem.202500956)
Supplement: Supplementary file 1 — Supporting Information [file CHEM-31-e202500956-s001.pdf]

Supporting Information

Differential Scanning Calorimetry........ S1
Powder X-ray Diffraction S2
Crystallography........ S5
Computational details........ S9

Differential Scanning Calorimetry

A Differential Scanning Calorimetry analysis was performed for **PAM·2QUI** obtained after grinding. Thermogram was recorded and elaborated using Pyris Software V12 (PerkinElmer). Endothermic and exothermic peaks were integrated, and all values are reported in J/g. A summary is reported in Table S1.

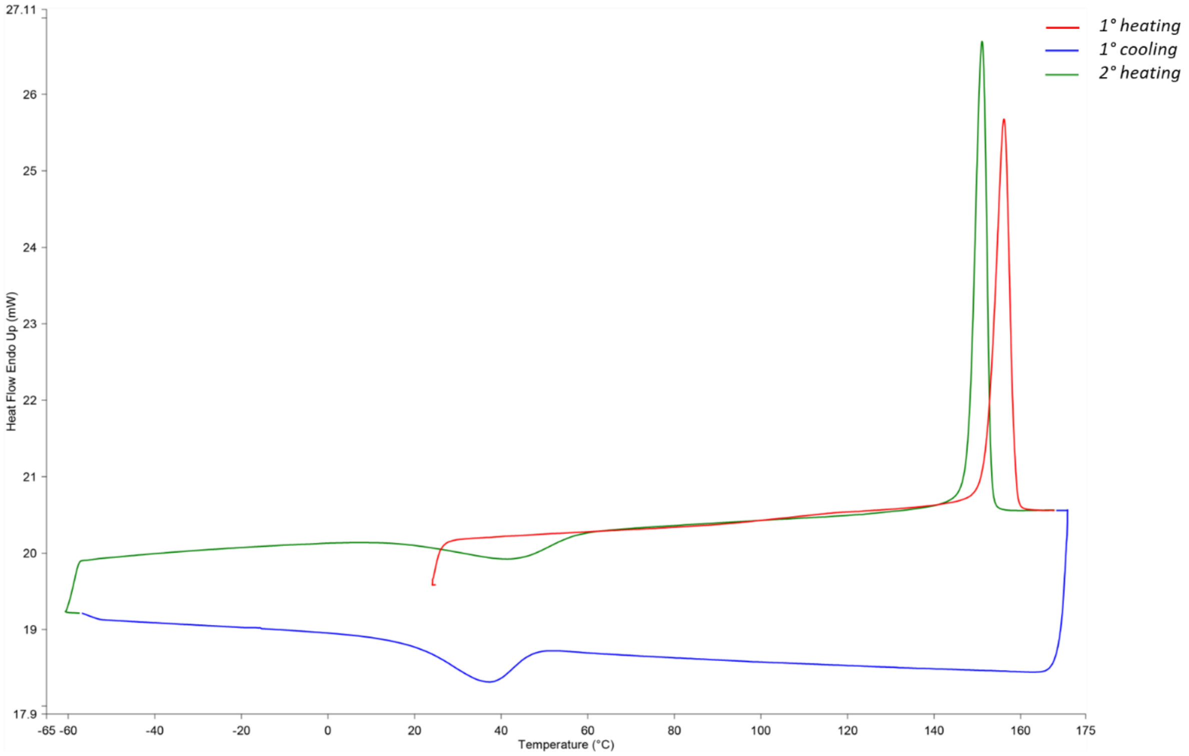

Figure S1. Thermogram of **PAM·2QUI**. First heating run from 20 °C to 170 °C (red curve), cooling run from 170 °C to -60 °C (blue curve) and second heating run from -60 °C to 170 °C (green curve). The whole firing profile was performed at 5°C/min.

Table S1. Summary of the thermal events that occurred during the DSC measurement of **PAM·2QUI**.

Compound	***Run***		***Thermal event***	***Temperature (°C)***	***ΔH (J/g)***
PAM·2QUI	First heating	1st peak	Endothermic	156.19	77.26
	First cooling	1st peak	Exothermic	36.80	-27.30
	Second heating	1st peak	Exothermic	42.23	-24.22
		2nd peak	Endothermic	151.08	69.40

Powder X-ray Diffraction

A Powder X-ray Diffraction (PXRD) analysis was performed for **PAM·2QUI** obtained after grinding in Bragg-Brentano geometry with Cu Kα radiation on a Rigaku SmartLab XE diffractometer equipped with a solid-state Hypix3000 2D detector. The sample was placed on a glass support and exposed to radiation (3 ° ≤ 2θ ≤ 30 °) with a scan of 20 °/min. Powder patterns were fitted by means of Pawley refinement using experimental cell parameters obtained by the single crystal X-ray experiment.

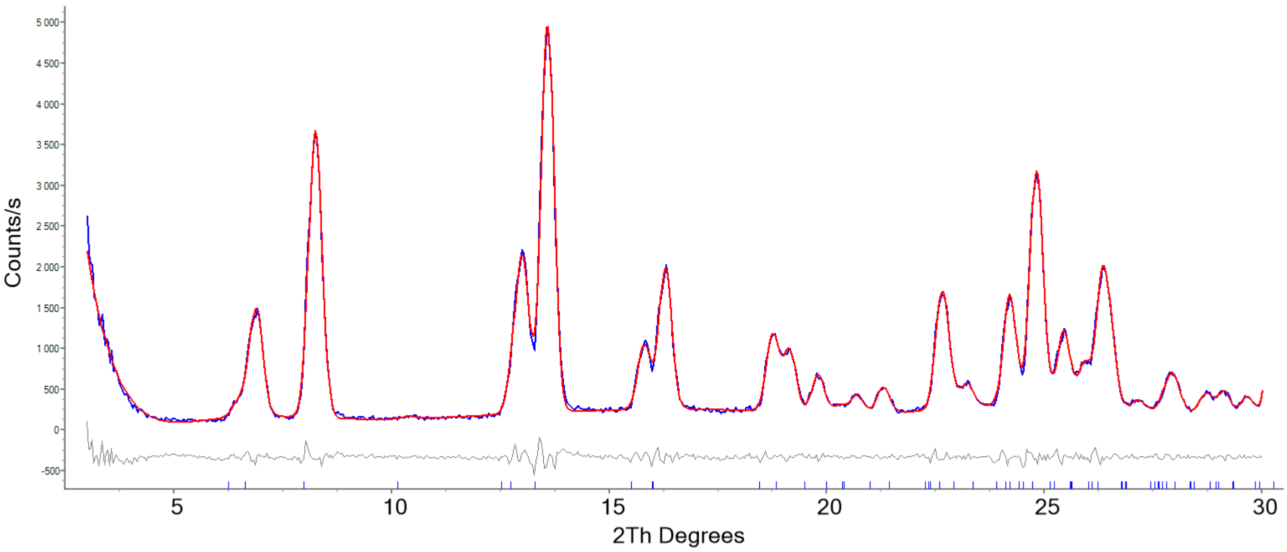

Figure S2. Pawley fit of **PAM·2QUI** (red line) against experimental data (blue line). Tick marks indicate calculated reflection positions. Y_{calc}-Y_{obs} residual curve is reported in grey.

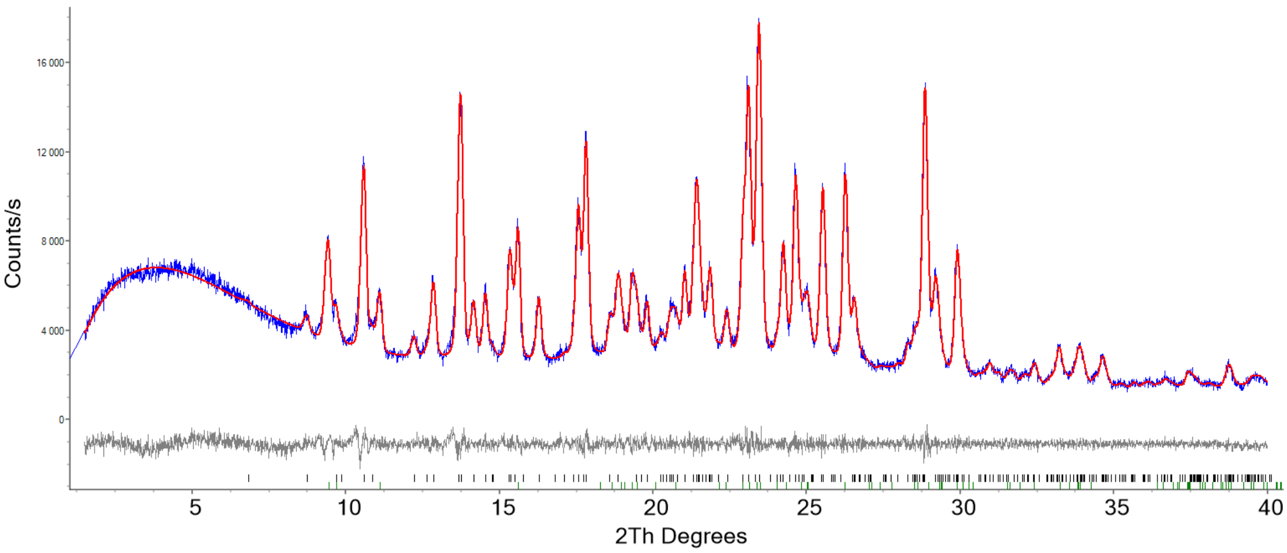

Figure S3. Pawley fit of **PAM·QUI·DMSO** (red line) against experimental data (blue line). A multi-phase refinement was performed since a residual amount of unreacted **PAM** was found. Tick marks indicate calculated reflection positions. Y_{calc}-Y_{obs} residual curve is reported in grey.

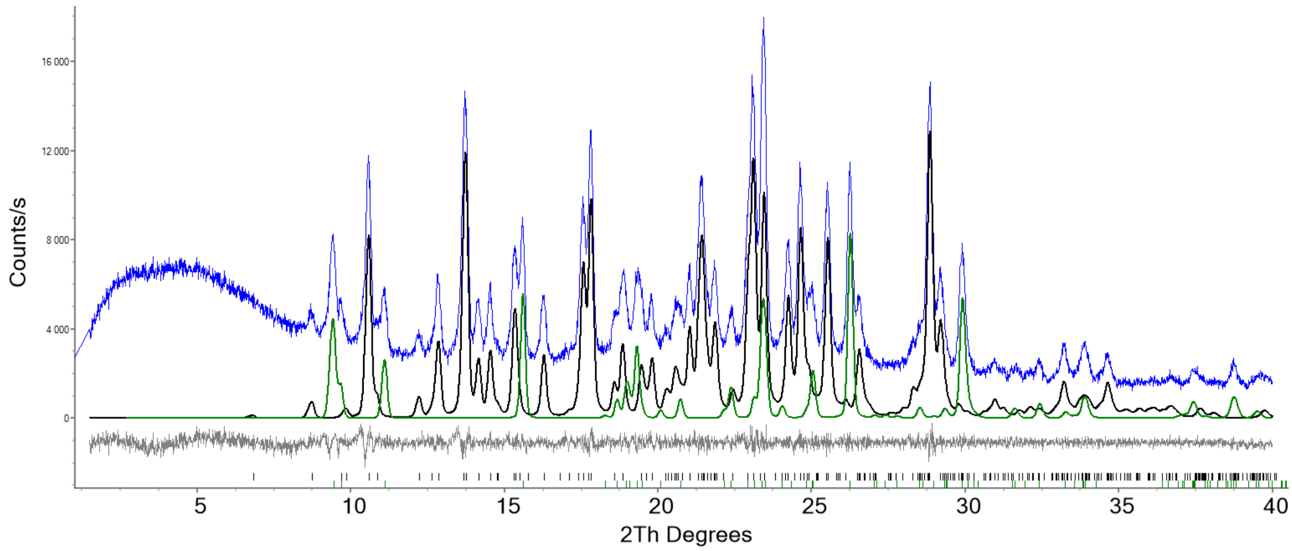

Figure S4. Plot of **PAM·QUI·DMSO** (black line) and **PAM** (green line) phases calculated in multi-phase Pawley refinement (Figure S3) against experimental data (blue line). Tick marks indicate calculated reflection positions. Y_{calc}-Y_{obs} residual curve is reported in grey.

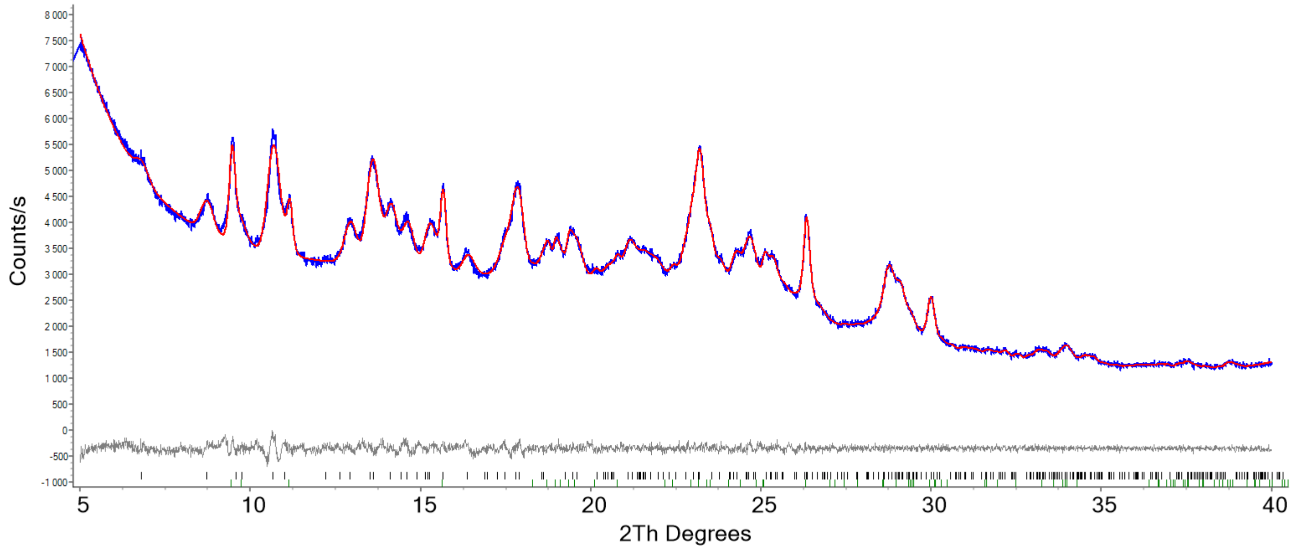

Figure S5. Pawley fit of **PAM·QUI·THF** (red line) against experimental data (blue line). A multi-phases refinement was performed since a residual amount of unreacted **PAM** was found. Tick marks indicate calculated reflection positions. Y_{calc}-Y_{obs} residual curve is reported in grey.

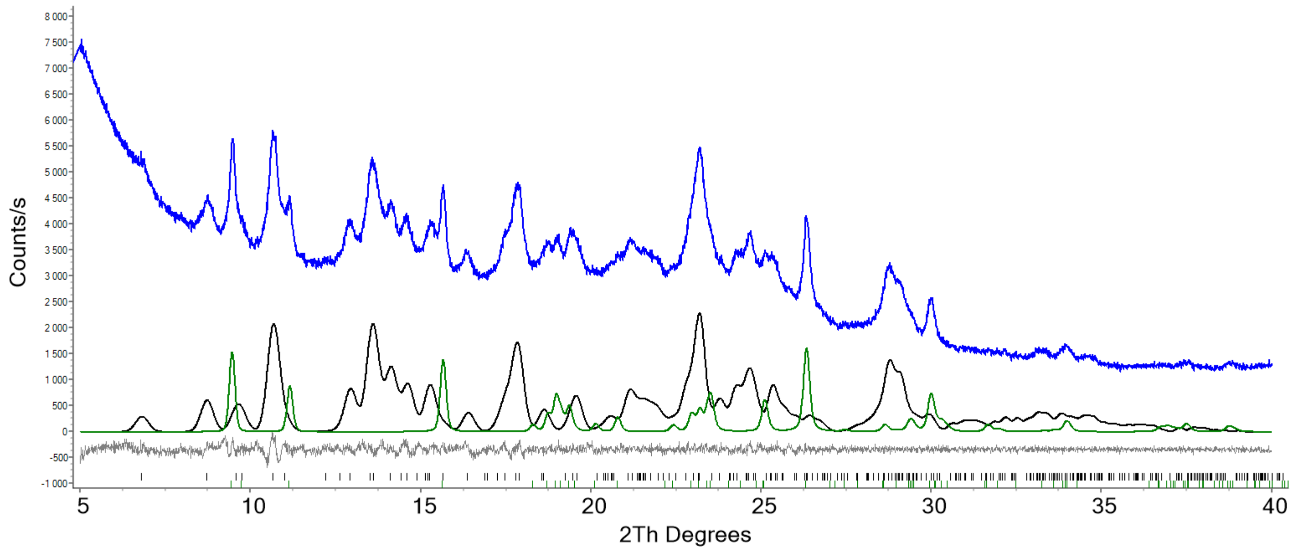

Figure S6. Plot of **PAM·QUI·THF** (black line) and **PAM** (green line) phases calculated in multi-phase Pawley refinement (Figure S5) against experimental data (blue line). Tick marks indicate calculated reflection positions. Y_{calc}-Y_{obs} residual curve is reported in grey.

Crystallography

Table S2. Crystallographic data and structure refinement of **PAM·2QUI**, **PAM·QUI·DMSO**, and **PAM·QUI·THF**.

Identification code	PAM·2QUI	PAM·QUI·DMSO	PAM·QUI·THF
Empirical formula	$C_{41}H_{30}N_2O_6$	$C_{34}H_{23}NO_7S$	$C_{36}H_{31}NO_7$
Temperature (K)	300	293	150
Crystal system	Monoclinic	Monoclinic	Monoclinic
Space group	C2/c	$P2_1/c$	$P2_1/c$
a (Å)	27.285 (4)	13.7878 (10)	13.7679 (6)
b (Å)	4.0264 (5)	12.5549 (10)	12.7995 (6)
c (Å)	28.928 (4)	17.3119 (14)	17.1202 (8)
α (°)	90	90	90
β (°)	103.546 (4)	110.651 (2)	111.411 (2)
γ (°)	90	90	90
Volume ($Å^3$)	3089.7 (7)	2804.2 (4)	2808.7 (2)
Z, Z'	4, 0.5	4, 1	4, 1
ρ_{calc} /g cm^{-3}	1.39	1.397	1.394
μ/mm^{-1}	0.094	0.169	0.791
Crystal size/mm^3	0.04×0.02×0.01	0.1×0.05×0.02	0.1×0.05×0.05
Radiation	MoKα (λ = 0.71073)	MoKα (λ = 0.71073)	CuKα (λ = 1.54178)
2θ range for data collection/°	4.69 to 43.93	4.104 to 51.398	6.896 to 140.346
Index ranges	-28 ≤ h ≤ 28, -4 ≤ k ≤ 4, -30 ≤ l ≤ 30	-16 ≤ h ≤ 15, -15 ≤ k ≤ 15, -21 ≤ l ≤ 21	-16 ≤ h ≤ 16, -15 ≤ k ≤ 15, -20 ≤ l ≤ 20
Reflections collected	8783	5328	44336
Independent reflections	1867 R_{int} = 0.0785, R_{sigma} = 0.0885	5328 R_{int} = 0.1007, R_{sigma} = 0.0483	5318 R_{int} = 0.0351, R_{sigma} = 0.0192
Data/restraints/parameters	1867/0/224	5328/4/408	5318/0/407
Goodness-of-fit on F^2	1.086	1.081	1.022
Final R indexes [I ≥ 2σ (I)]	R_1 = 0.1309, wR_2 = 0.2967	R_1 = 0.0758, wR_2 = 0.2133	R_1 = 0.0684, wR_2 = 0.2026
Final R indexes [all data]	R_1 = 0.2312, wR_2 = 0.3860	R_1 = 0.1116, wR_2 = 0.2358	R_1 = 0.0779, wR_2 = 0.2132
Largest diff. peak/hole/ e $Å^{-3}$	0.52/-0.41	0.65/-0.40	0.85/-0.69
CCDC deposition number	2429997	2429995	2429996

Table S3. Report of IUCR checkCIF service tool for **PAM·2QUI.**

Datablock: PAM_2QUI

Bond precision: C-C = 0.0155 A Wavelength=0.71073

Cell:	a=27.285(4)	b=4.0264(5)	c=28.928(4)
	alpha=90	beta=103.546(4)	gamma=90
Temperature:	300 K		

	Calculated	Reported
Volume	3089.6(7)	3089.7(7)
Space group	C 2/c	C 1 2/c 1
Hall group	-C 2yc	-C 2yc
Moiety formula	C23 H16 O6, 2(C9 H7 N)	C23 H16 O6, 2(C9 H7 N)
Sum formula	C41 H30 N2 O6	C41 H30 N2 O6
Mr	646.67	646.67
Dx,g cm-3	1.390	1.390
Z	4	4
Mu (mm-1)	0.094	0.094
F000	1352.0	1352.0
F000'	1352.65	
h,k,lmax	28,4,30	28,4,30
Nref	1894	1867
Tmin,Tmax	0.998,0.999	0.598,0.745
Tmin'	0.996	

Correction method= # Reported T Limits: Tmin=0.598 Tmax=0.745
AbsCorr = MULTI-SCAN

Data completeness= 0.986 Theta(max)= 21.965

R(reflections)= 0.1309(741) wR2(reflections)= 0.3860(1867)

S = 1.086 Npar= 224

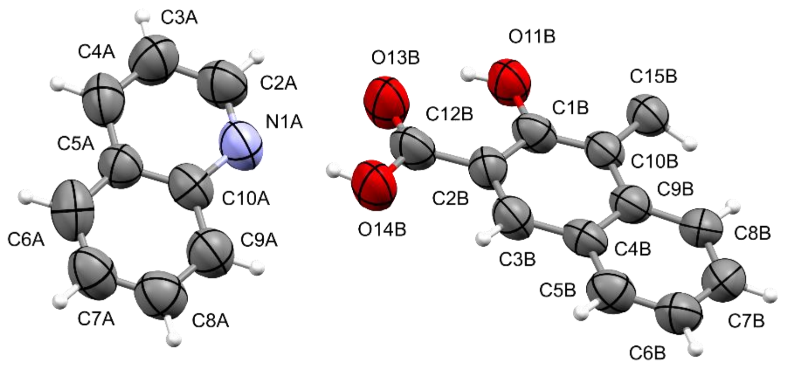

Figure S7. ORTEP drawing of **PAM·2QUI**. All non-hydrogen atoms shown as ellipsoids at the 50% probability level. H atoms (isotropically refined) are reported in ball-and-stick style for sake of clarity. Labels contain suffixes (A, B) for each independent molecule in the asymmetric unit. Color code: grey=C, red=O, blue=N, white=H.

Table S4. Report of IUCR checkCIF service tool for **PAM·QUI·DMSO**.

Datablock: PAM_QUI_DMSO

Bond precision:	C-C = 0.0045 A		Wavelength=0.71073
Cell:	a=13.7878(10)	b=12.5549(10)	c=17.3119(14)
	alpha=90	beta=110.651(2)	gamma=90
Temperature:	293 K		

	Calculated	Reported
Volume	2804.2(4)	2804.2(4)
Space group	P 21/c	P 1 21/c 1
Hall group	-P 2ybc	-P 2ybc
Moiety formula	C23 H15 O6, C9 H8 N, C2 O S	C23 H15 O6, C9 H8 N, C2 O S
Sum formula	C34 H23 N O7 S	C34 H23 N O7 S
Mr	589.59	589.59
Dx,g cm-3	1.397	1.397
Z	4	4
Mu (mm-1)	0.169	0.169
F000	1224.0	1224.0
F000'	1225.12	
h,k,lmax	16,15,21	0,0,0
Nref	5334	5328
Tmin,Tmax	0.990,0.997	
Tmin'	0.983	

Correction method= Not given

Data completeness= 0.999 Theta(max)= 25.699

R(reflections)= 0.0758(3621) wR2(reflections)= 0.2358(5328)

S = 1.081 Npar= 408

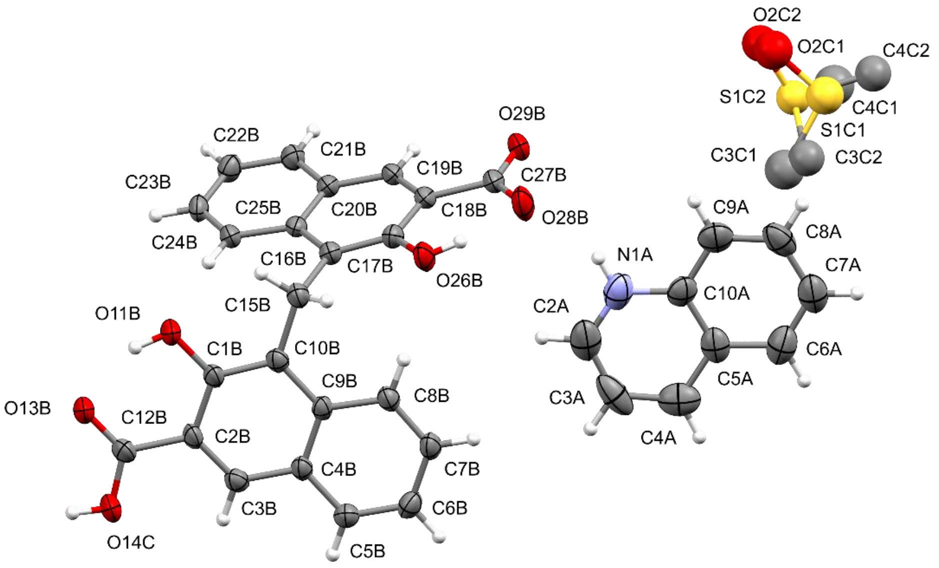

Figure S8. ORTEP drawing of **PAM·QUI·DMSO**. All non-hydrogen atoms shown as ellipsoids at the 50% probability level. H atoms (isotropically refined) are reported in ball-and-stick style for sake of clarity. Due to extensive disorder, thermal parameters and atom positions of DMSO molecule could not be refined reliably. Therefore, DMSO molecule is reported as isotropically refined without H atoms. Labels contain suffixes (A, B, C) for each independent molecule in the asymmetric unit. Color code: grey=C, red=O, blue=N, white=H.

Table S5. Report of IUCR checkCIF service tool for **PAM·QUI·THF**.

Datablock: PAM_QUI_THF

Bond precision:	C-C = 0.0042 A		Wavelength=1.54178
Cell:	a=13.7679(6)	b=12.7995(6)	c=17.1202(8)
	alpha=90	beta=111.411(2)	gamma=90
Temperature:	150 K		

	Calculated	Reported
Volume	2808.8(2)	2808.7(2)
Space group	P 21/c	P 1 21/c 1
Hall group	-P 2ybc	-P 2ybc
Moiety formula	C23 H15 O6, C9 H8 N, C4 H8 O	C23 H15 O6, C9 H8 N, C4 H8 O
Sum formula	C36 H31 N O7	C36 H31 N O7
Mr	589.62	589.62
Dx,g cm-3	1.394	1.394
Z	4	4
Mu (mm-1)	0.791	0.791
F000	1240.0	1240.0
F000'	1243.95	
h,k,lmax	16,15,20	16,15,20
Nref	5342	5318
Tmin,Tmax	0.752,0.854	0.687,0.753
Tmin'	0.673	

Correction method= # Reported T Limits: Tmin=0.687 Tmax=0.753
AbsCorr = MULTI-SCAN

Data completeness= 0.996 Theta(max)= 70.173

R(reflections)= 0.0684(4621) wR2(reflections)= 0.2132(5318)

S = 1.022 Npar= 407

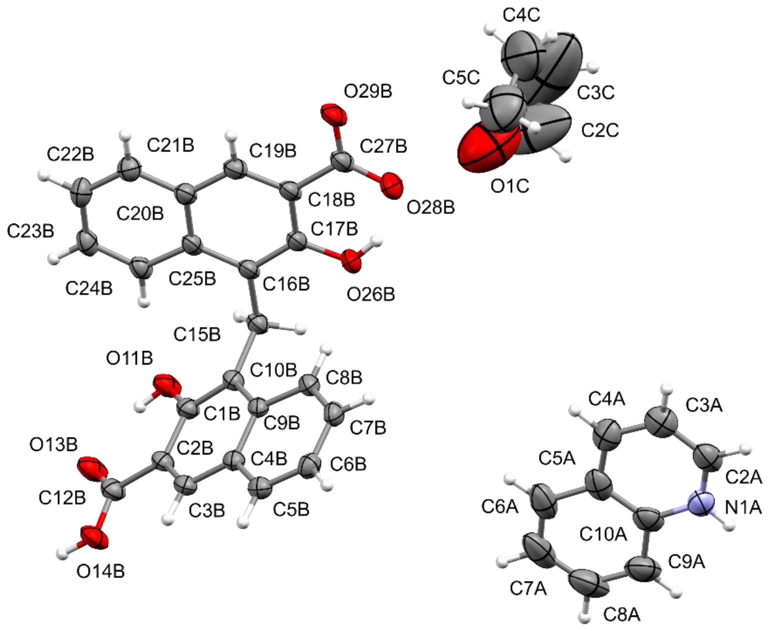

Figure S9. ORTEP drawing of **PAM·QUI·THF**. All non-hydrogen atoms shown as ellipsoids at the 50% probability level. H atoms (isotropically refined) are reported in ball-and-stick style for sake of clarity. Labels contain suffixes (A, B, C) for each independent molecule in the asymmetric unit. Color code: grey=C, red=O, blue=N, white=H.

Computational details

Table S6. Cartesian coordinates for the simulated pamoic acid and pamoate anion.

Pamoic Acid					Pamoate Anion			
**O**	-0.96064	2.227513	-0.12248		**O**	-2.41989	1.481749	1.667261
**H**	-1.43828	2.957155	0.332633		**H**	-3.34682	1.92	1.430547
**O**	-4.72051	2.486322	1.607324		**O**	2.356265	-1.47278	1.687186
**H**	-4.89705	3.376815	1.949811		**H**	3.224247	-1.91554	1.566444
**O**	-2.81908	3.513086	1.112862		**O**	-5.80802	0.563615	-0.48517
**C**	-1.31816	-0.03067	-0.67128		**O**	5.699464	-0.57118	-0.57303
**C**	-3.04699	1.233347	0.529415		**H**	6.412386	-1.21784	-0.44891
**C**	-3.44199	-1.10154	-0.02583		**O**	4.730634	-1.96128	0.870036
**C**	-2.16512	-1.16906	-0.66452		**O**	-4.70994	2.063667	0.734895
**C**	-1.82827	-2.4068	-1.27483		**C**	-1.30354	-1.58871	0.03221
**H**	-0.85925	-2.52507	-1.74138		**C**	2.39405	1.915495	-0.73544
**H**	-0.03478	0.866478	-2.08635		**C**	3.500502	1.040925	-0.70597
**C**	-1.76286	1.135961	-0.0826		**H**	4.379122	1.281001	-1.29871
**C**	-3.85904	0.114281	0.569278		**C**	2.341423	-0.39379	0.870072
**H**	-4.83619	0.169373	1.039617		**C**	-2.46957	-1.92423	-0.71848
**C**	-2.67968	-3.48863	-1.24268		**C**	-1.26208	-0.38381	0.797112
**H**	-2.38041	-4.42191	-1.71381		**C**	1.232675	1.588634	0.02621
**C**	-3.51247	2.500707	1.108842		**C**	1.194883	0.38815	0.798744
**C**	-4.29912	-2.23003	-0.01052		**C**	-2.40638	0.403448	0.854017
**H**	-5.26311	-2.13629	0.48658		**C**	3.501229	-0.08018	0.093201
**C**	-3.93399	-3.39332	-0.60247		**C**	-0.03481	0.004202	1.603937
**H**	-4.59915	-4.25394	-0.58582		**H**	0.244589	-0.81497	2.273926
**O**	0.960629	-2.22752	-0.1225		**H**	-0.31843	0.844666	2.245999
**H**	1.438805	-2.95724	0.332533		**C**	-3.57184	0.083598	0.089516
**O**	4.720491	-2.48635	1.607372		**C**	-0.21929	-2.5137	0.009704
**H**	4.897082	-3.37669	1.950246		**H**	0.666739	-2.32588	0.601886
**O**	2.819056	-3.51309	1.112847		**C**	-3.58084	-1.05284	-0.68558
**C**	1.318139	0.030684	-0.67128		**H**	-4.49058	-1.26511	-1.24184
**C**	3.046998	-1.23336	0.529359		**C**	2.444737	3.127719	-1.47315
**C**	3.442035	1.10156	-0.02584		**H**	3.342868	3.344965	-2.05011
**C**	2.165102	1.169062	-0.66452		**C**	0.162068	2.525055	0.022559
**C**	1.828252	2.406813	-1.27482		**H**	-0.73231	2.329632	0.606597
**H**	0.859227	2.52509	-1.74137		**C**	4.701196	-0.95597	0.158777
**H**	0.03484	-0.86642	-2.08637		**C**	0.258051	3.707213	-0.67408
**C**	1.762849	-1.13596	-0.08262		**H**	-0.5733	4.408307	-0.6388
**C**	3.859008	-0.1143	0.56926		**C**	1.400461	4.018085	-1.43823
**H**	4.836147	-0.16943	1.039616		**H**	1.452337	4.952619	-1.99287
**C**	2.679659	3.488634	-1.24266		**C**	-4.78615	0.959196	0.118425
**H**	2.380409	4.421921	-1.71379		**C**	-0.2991	-3.68363	-0.717
**C**	3.512475	-2.50074	1.108761		**H**	0.53988	-4.37886	-0.70659
**C**	4.299114	2.230026	-0.01053		**C**	-2.51933	-3.13562	-1.45547
**H**	5.263108	2.136278	0.486556		**H**	-3.42761	-3.3623	-2.01192
**C**	3.933996	3.393332	-0.60246		**C**	-1.45513	-3.99947	-1.4581
**H**	4.599154	4.253948	-0.58582		**H**	-1.50065	-4.92979	-2.02302
**C**	0.000037	0.000025	-1.41983					

Table S7. Cartesian Coordinates for the simulated quinoline and quinolinium cation**.**

Quinoline					Quinolinium Cation			
**N**	1.168723	-1.41426	0.015728		**N**	1.200502	-1.30847	0.005671
**C**	0.014032	-0.68522	-0.00787		**H**	1.206199	-2.32456	0.006648
**C**	0.022083	0.709258	-0.00152		**C**	-0.02676	0.741515	0.006808
**C**	-1.23093	-1.40278	-0.01154		**C**	-0.00805	-0.67607	0.011106
**H**	-1.19342	-2.48751	-0.02106		**C**	2.342943	-0.66287	-0.01482
**C**	-1.20783	1.403084	0.009338		**H**	3.243322	-1.27002	-0.02897
**H**	-1.20713	2.491266	0.021504		**C**	1.214611	1.420307	0.018112
**C**	2.315544	-0.77151	0.012823		**H**	1.222859	2.507724	0.030696
**H**	3.219202	-1.38267	0.031563		**C**	2.390295	0.715094	-0.00995
**C**	-2.39382	-0.69031	-0.01554		**H**	3.354804	1.210754	-0.01816
**H**	-3.34951	-1.21114	-0.0231		**C**	-1.20492	-1.40415	0.005966
**C**	-2.37867	0.701888	0.020768		**H**	-1.19025	-2.49149	0.008634
**H**	-3.32442	1.240558	0.035761		**C**	-2.433	0.681597	-0.01166
**C**	1.272563	1.377994	-0.01979		**H**	-3.39518	1.18502	-0.02229
**H**	1.297304	2.465894	-0.03066		**C**	-1.26689	1.416696	-0.00387
**C**	2.417006	0.636814	-0.00534		**H**	-1.28113	2.502759	-0.00734
**H**	3.39704	1.108156	-0.01214		**C**	-2.38287	-0.71372	-0.00217
					**H**	-3.3163	-1.2713	-0.00601

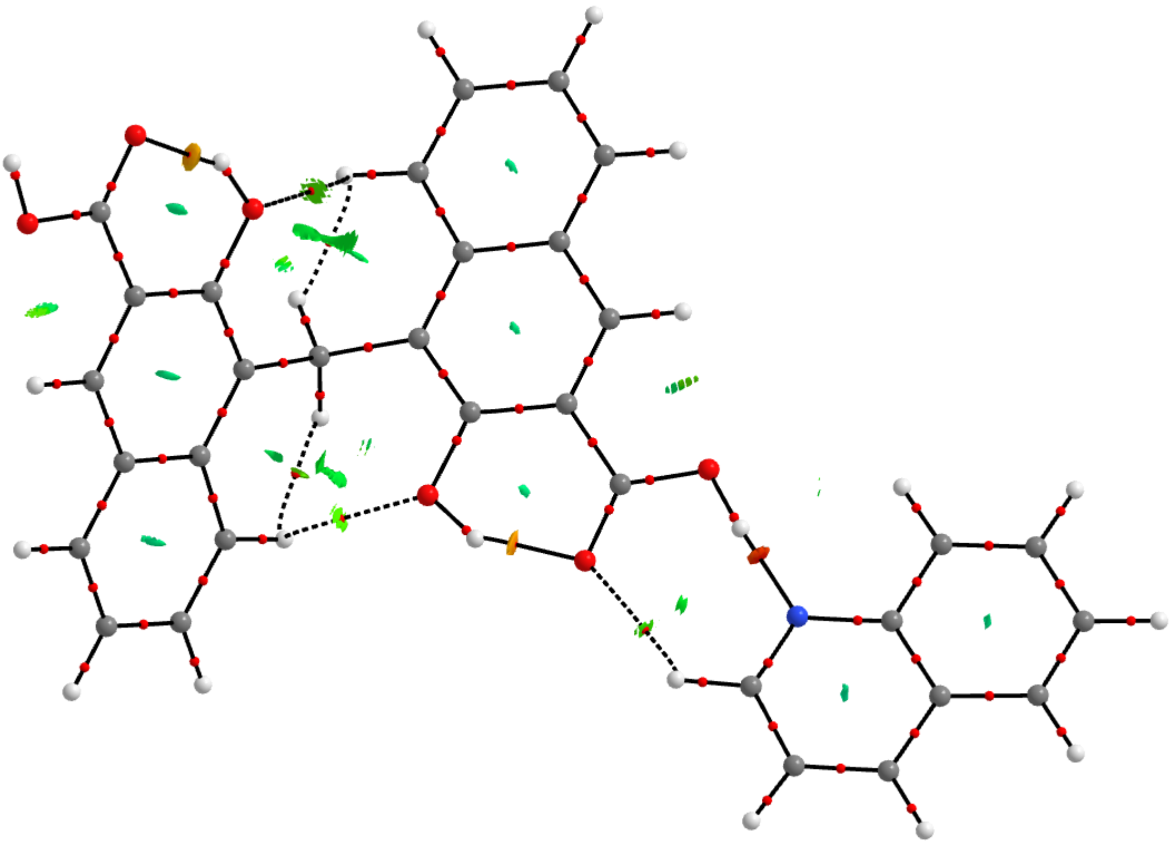

Figure S10. Details of the QTAIM and NCIPlot as plotted by the program AIMAll for the **PAM**·**QUI** hydrogen bonded dimer as present in **PAM·2QUI**. Bond Critical Points (BCPs) are red spheres, and the bond paths are black lines. NCIPlot surfaces (RDG= 0.3 a.u., ρ= 0.1 a.u., colour range ±0.3 a.u.) are superimposed to the structure.

Table S8. SAPT0 interaction energies for the **PAM**·**QUI** hydrogen bonded dimer as present in **PAM·2QUI** as retried from the Psi4 program. All the values are in kcal/mol.

**Interaction Energy**	-16.765
**Electrostatics**	-27.447
**Exchange**	32.331
**Induction**	-14.378
**Dispersion**	-7.271

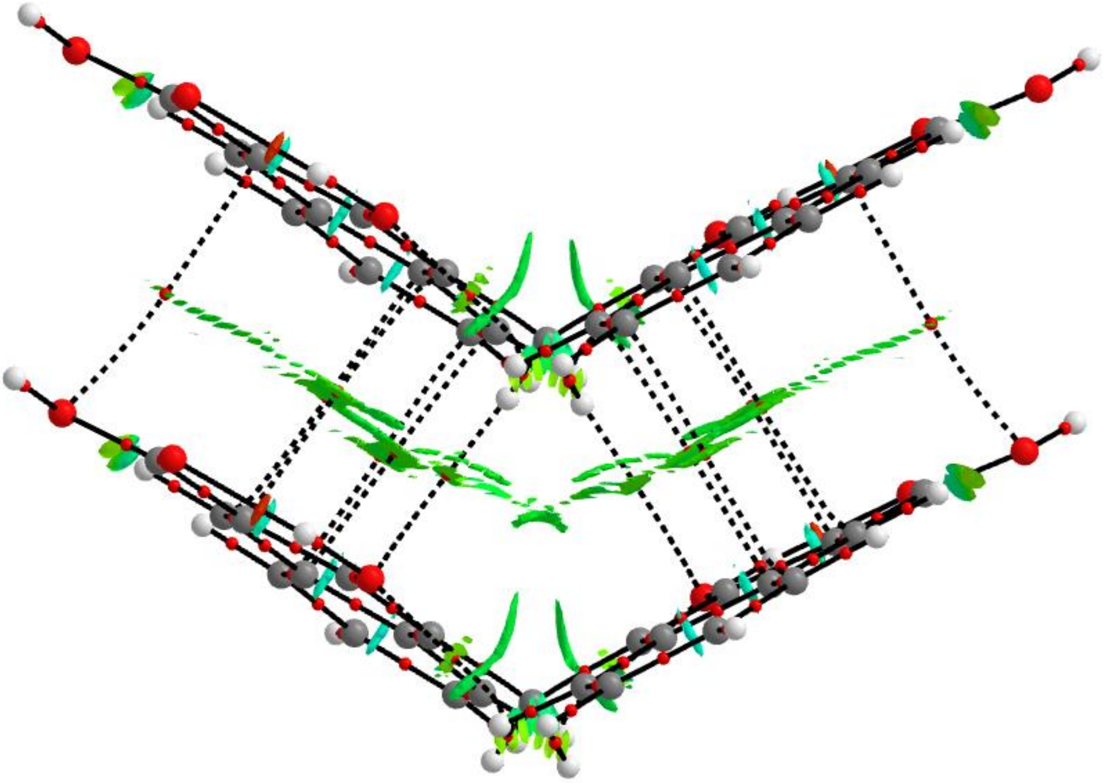

Figure S11. Details of the QTAIM and NCIPlot as plotted by the program AIMAll for the **PAM·PAM** π-π dimer as present in **PAM·2QUI**. Bond Critical Points (BCPs) are red spheres, and the bond paths are black lines. NCIPlot surfaces (RDG= 0.3 a.u., ρ= 0.1 a.u., colour range ±0.3 a.u.) are superimposed to the structure.

Table S9. SAPT0 interaction energies for the **PAM·PAM** π-π dimer as present in **PAM·2QUI** as retried from the Psi4 program. All the values are in kcal/mol.

**Interaction Energy**	-25.305
**Electrostatics**	-9.425
**Exchange**	26.167
**Induction**	-2.776
**Dispersion**	-39.272

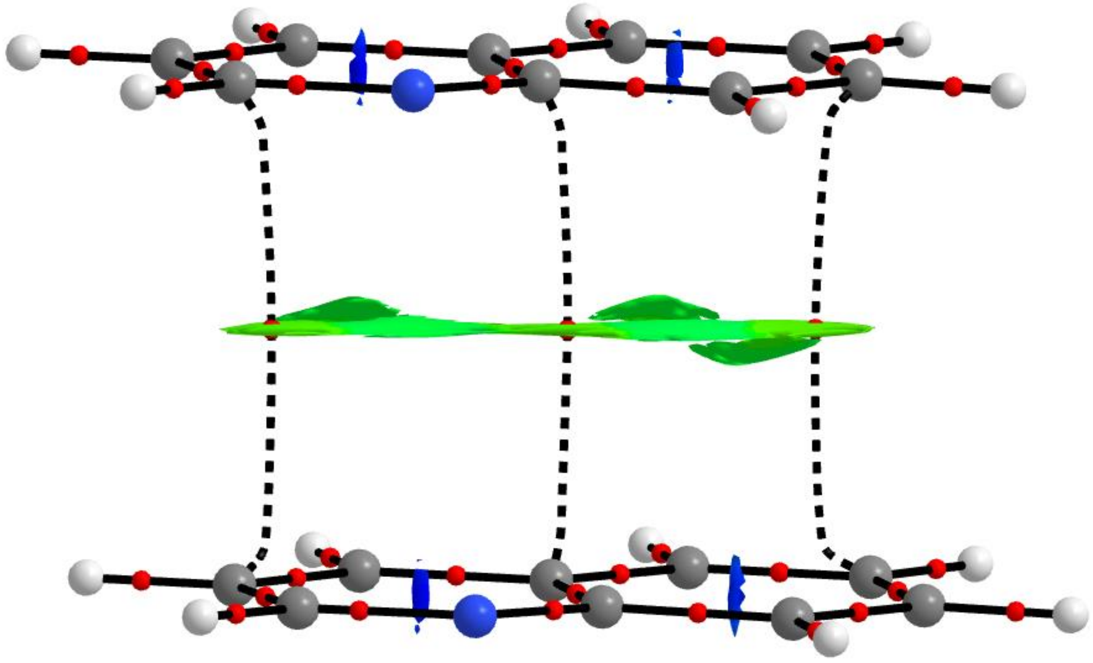

Figure S12. Details of the QTAIM and NCIPlot as plotted by the program AIMAll for the **QUI·QUI** π-π dimer as present in **PAM·2QUI**. Bond Critical Points (BCPs) are red spheres, and the bond paths are black lines. NCIPlot surfaces (RDG= 0.3 a.u., ρ= 0.1 a.u., colour range ±0.3 a.u.) are superimposed to the structure.

Table S10. SAPT0 interaction energies for the **QUI·QUI** π-π dimer as present in **PAM·2QUI** as retried from the Psi4 program. All the values are in kcal/mol.

**Interaction Energy**	-16.765
**Electrostatics**	-1.34258
**Exchange**	6.120
**Induction**	-0.577
**Dispersion**	-10.200

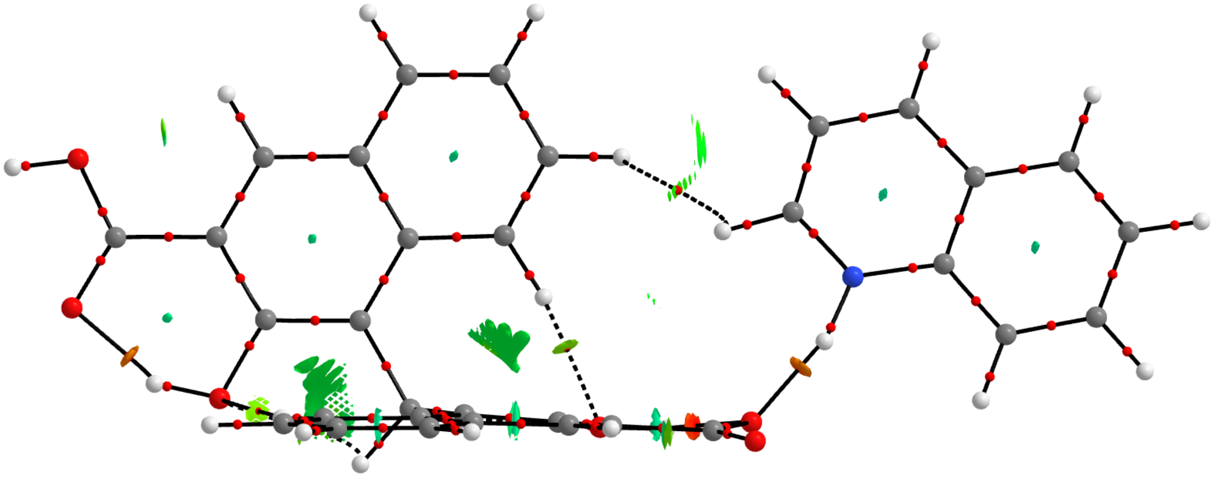

Figure S13. Details of the QTAIM and NCIPlot as plotted by the program AIMAll for the **PAM**·**QUI** hydrogen bonded dimer as present in **PAM·QUI·THF**. Bond Critical Points (BCPs) are red spheres, and the bond paths are black lines. NCIPlot surfaces (RDG= 0.3 a.u., ρ= 0.1 a.u., colour range ±0.3 a.u.) are superimposed to the structure.

Table S11. SAPT0 interaction energies for the **PAM**·**QUI** hydrogen bonded dimer as present in **PAM·QUI·THF** as retried from the Psi4 program. All the values are in kcal/mol.

**Interaction Energy**	-93.599
**Electrostatics**	-85.400
**Exchange**	22.017
**Induction**	-22.904
**Dispersion**	-7.312

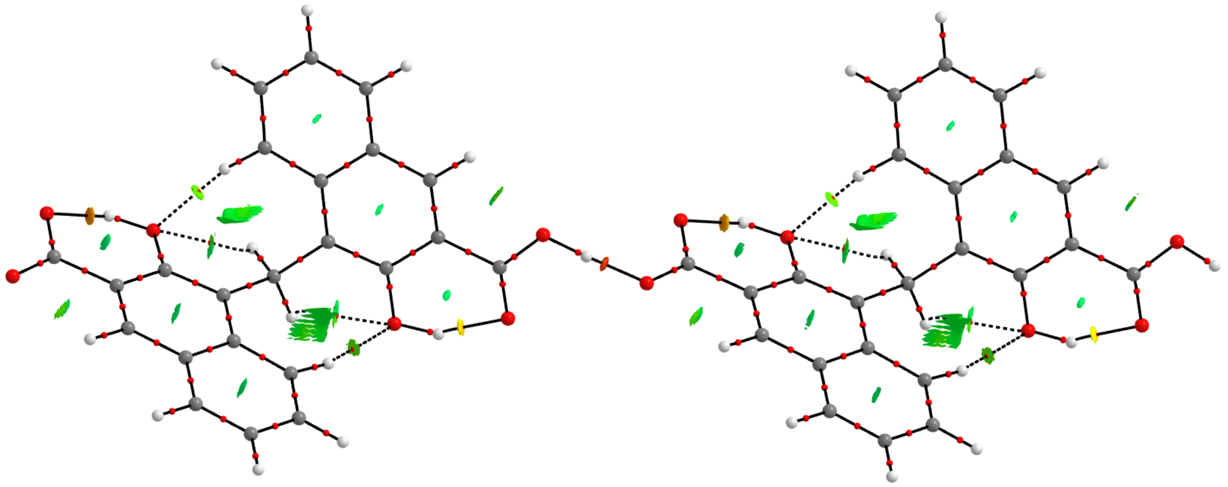

Figure S14. Details of the QTAIM and NCIPlot as plotted by the program AIMAll for the **PAM**·**PAM** hydrogen bonded dimer as present in **PAM·QUI·THF**. Bond Critical Points (BCPs) are red spheres, and the bond paths are black lines. NCIPlot surfaces (RDG= 0.3 a.u., ρ= 0.1 a.u., colour range ±0.3 a.u.) are superimposed to the structure.

Table S12. SAPT0 interaction energies for the **PAM**·**PAM** hydrogen bonded dimer as present in **PAM·QUI·THF** as retried from the Psi4 program. All the values are in kcal/mol.

**Interaction Energy**	-3.987
**Electrostatics**	-12.199
**Exchange**	39.863
**Induction**	-25.258
**Dispersion**	-6.394

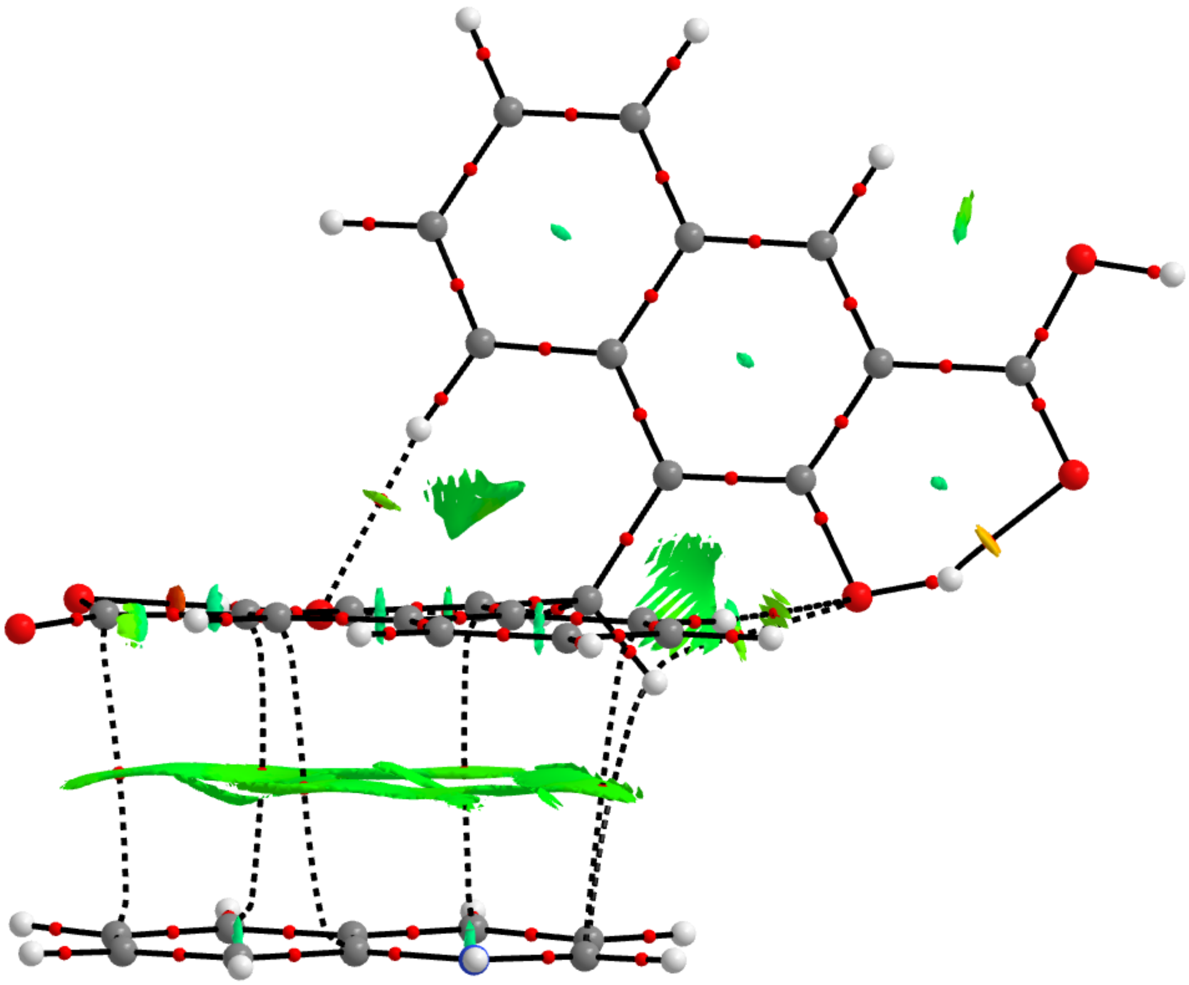

Figure S15. Details of the QTAIM and NCIPlot as plotted by the program AIMAll for the first **PAM·QUI** π-π dimer as present in **PAM·QUI·THF**. Bond Critical Points (BCPs) are red spheres, and the bond paths are black lines. NCIPlot surfaces (RDG= 0.3 a.u., ρ= 0.1 a.u., colour range ±0.3 a.u.) are superimposed to the structure.

Table S13. SAPT0 interaction energies for the first **PAM·QUI** π-π dimer as present in **PAM·QUI·THF** as retried from the Psi4 program. All the values are in kcal/mol.

**Interaction Energy**	-82.251
**Electrostatics**	-70.324
**Exchange**	16.591
**Induction**	-7.813
**Dispersion**	-20.704

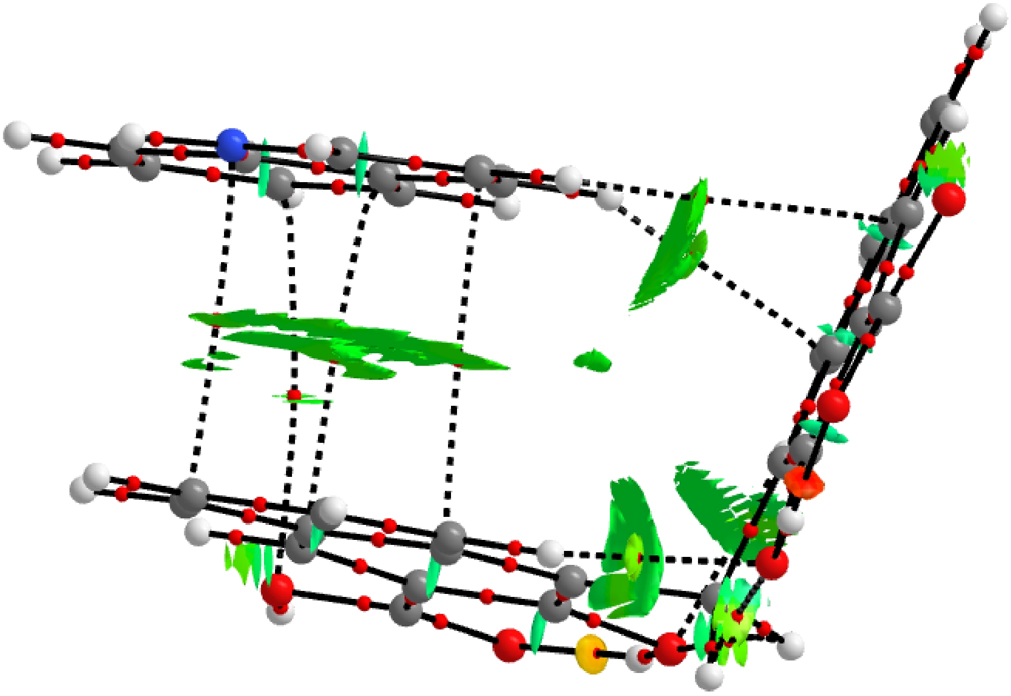

Figure S16. Details of the QTAIM and NCIPlot as plotted by the program AIMAll for the second **PAM·QUI** π-π dimer as present in **PAM·QUI·THF**. Bond Critical Points (BCPs) are red spheres, and the bond paths are black lines. NCIPlot surfaces (RDG= 0.3 a.u., ρ= 0.1 a.u., colour range ±0.3 a.u.) are superimposed to the structure.

Table S14. SAPT0 interaction energies for the second **PAM·QUI** π-π dimer as present in **PAM·QUI·THF** as retried from the Psi4 program. All the values are in kcal/mol.

**Interaction Energy**	-70.876
**Electrostatics**	-56.942
**Exchange**	15.738
**Induction**	-8.898
**Dispersion**	-20.774

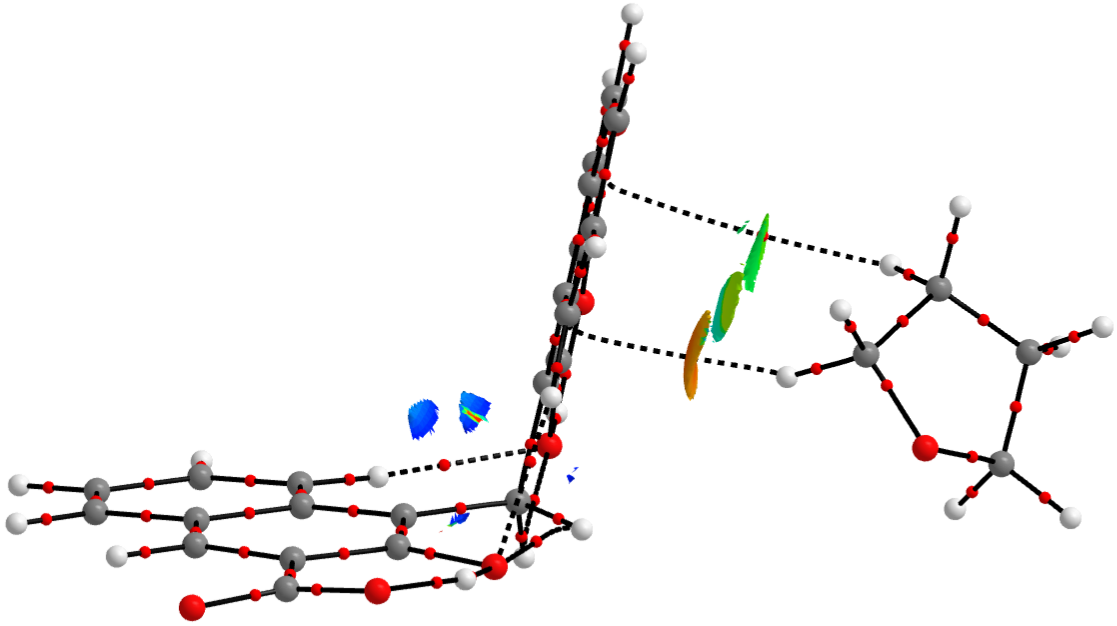

Figure S17. Details of the QTAIM and NCIPlot as plotted by the program AIMAll for the second **PAM**·THF dimer as present in **PAM·QUI·THF**. Bond Critical Points (BCPs) are red spheres, and the bond paths are black lines. NCIPlot surfaces (RDG= 0.3 a.u., ρ= 0.1 a.u., colour range ±0.3 a.u.) are superimposed to the structure.

Table S15. SAPT0 interaction energies for **PAM**-THF dimer as present in **PAM·QUI·THF** as retried from the Psi4 program. All the values are in kcal/mol.

**Interaction Energy**	-2.547
**Electrostatics**	-0.122
**Exchange**	2.888
**Induction**	-0.606
**Dispersion**	-4.709

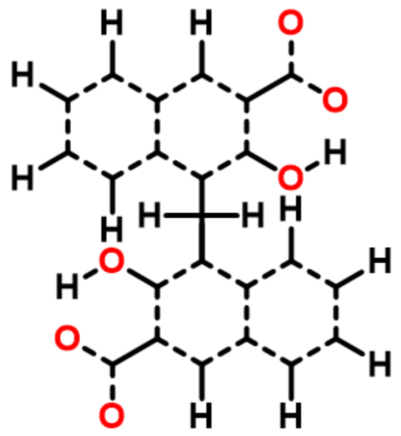

Figure S18. 3D setting employed in the CSD survey *via* Conquest. Dotted lines represent "any bond". Aromatic carbon atoms were additionally restricted to be bound to only three other atoms.

Table S16. Selected geometrical parameters extracted from the structures containing **PAM** present on the CSD. Solvates and pamoic acid crystal structures were excluded from the survey. In six hits (KAQBUB, KAQCAI, KAQCEM, KAQCIQ, TAPDUL, TAPFEX) pamoic acid is seemingly present in form A, but the conformer is not refined, so that many geometrical parameters could not be extracted. Lastly, five hits (DAZYOV, DAZYUB, DEFZEV, DEFZEV01, XEMYIY) are pamoate esters structures and were excluded from the survey.

Refcode	Form	HB - N	HB - O	π - π Hetero	π - π Homo	CH - π Hetero	CH - π Homo
**BEVXAD**	A	xx	2 460	3 594	xx	3 250	xx
**BEVXEH**	A	2 698	2 519	3 815	xx	xx	3 764
**BEVXIL**	A	2 667	2.593	3 304	xx	3 406	xx
**BEVXOR**	A	2 682	2.589	3 186	xx	3 541	xx
**BEVXUX**	B	2 605	xx	3 286	xx	3 595	xx
**BEVYAE**	A	2 835	2 462	3 358	xx	xx	xx
**DANGUY**	A	xx	2 462	xx	xx	3 508	3 753
**DANHEJ**	A	xx	2 456	3 495	3 504	3 632	3 804
**DANHIN**	A	xx	2 457	3 513	3 580	3 617	3 698
**DAZYOV**	Pamoate ester						
**DAZYUB**	Pamoate ester						
**DEFZEV**	Pamoate ester						
**DEFZEV01**	Pamoate ester						
**DEGDAV**	Pamoic acid (not solvated)						
**DEGDAV01**	Pamoic Acid (not solvated)						
**DEXBUF**	B	2 738	xx	3 529	xx	3 554	xx
**DEXBUF01**	B	2 767	xx	3 623	xx	3 591	xx
**HOQKUU**	B	2 566	xx	3 463	xx	3 680	xx
**HOQLAB**	B	2 531	xx	3 440	xx	3 538	xx
**HOQLEF**	B	2 558	xx	3 992	xx	3 881	xx
**JAWXEL**	B	2 571	xx	3 498	xx	3 481	xx
**JAWXIP**	A	2 696	2 521	3 377	xx	xx	xx
**JAWXOV**	A	2 758	2 496	3 396	xx	3 754	xx
**JAWXUB**	A	2 629	2 481	3 395	xx	3 600	xx

**JAWYAI**	A	2 664	2 497	3 257	xx	3 924	3 804
**JAWYEM**	No coordinates available						
**KAPZUY**	A	2 696	2 595	3 284	xx	3 555	xx
**KAQBAH**	A	2 684	2 596	3 360	xx	3 868	xx
**KAQBEL**	A	2 687	2 596	3 295	xx	3 572	xx
**KAQBIP**	A	2 685	2 587	3 432	xx	3 553	xx
**KAQBOV**	A	2 684	2 599	3 448	xx	3 555	xx
**KAQBUB**	Coformer not refined						
**KAQCAI**	Coformer not refined						
**KAQCEM**	Coformer not refined						
**KAQCIQ**	Coformer not refined						
**MOXROH**	B	xx	2 652	xx	xx	3 527	3 750
**MOXRUN**	C	2 585	xx	3 341	3 500	3 795	xx
**NIVCAZ**	A	2 658	xx	3 471	3 639	3 595	xx
**NIVCED**	A	2 654	xx	3 492	xx	3 848	xx
**NIVCIH**	A	2 654	xx	3 592	xx	3 691	xx
**NIVCON**	A	2 638	xx	3 549	xx	3 617	xx
**NIVCUT**	A	2 635	xx	3 481	xx	3 669	xx
**NIVDAA**	A	2 646	xx	3 454	xx	3 461	xx
**QEXJEJ**	A	xx	2 458	3 388	xx	3 512	xx
**QEXJEJ01**	A	xx	2 459	3 384	3 448	xx	3 687
**QEXJEJ02**	A	xx	2 451	3 471	3 403	3 488	3 852
**QIPNUC**	B	2 797	xx	xx	xx	xx	xx
**QIPNUC01**	B	2 985	xx	xx	xx	xx	xx
**QIPNUC02**	B	2 753	xx	xx	xx	xx	xx
**QIPPEO**	A	2 786	xx	xx	xx	xx	xx
**QIPPOY**	A	2 738	xx	xx	xx	3 782	xx
**QQQHBD**	No coordinates available						
**REJKID**	A	2 933	2 452	3 349	xx	3 563	xx
**SEFYIN**	B	2 539	xx	xx	3 471	3 532	xx
**SEFYOT**	B	2 657	2 688	xx	xx	3 422	xx
**SEFYUZ**	B	2 731	xx	xx	3 460	3 552	xx
**SIQCIF**	Pamoic acid (DMF solvated)						
**TABMAK**	A	2 533	xx	3 489	xx	3 586	xx
**TAPCIY**	A	2 891	2 496	3 443	xx	3 630	xx
**TAPCOE**	B	2 575	xx	3 450	xx	3 442	xx
**TAPCUK**	A	2 753	2 451	3 569	xx	3 398	xx
**TAPDAR**	A	xx	2 445	3 480	xx	3 285	xx
**TAPDEV**	A	xx	2 450	3 319	xx	3 898	xx
**TAPDIZ**	A	xx	2 457	3 352	xx	3 439	xx
**TAPDOF**	A	2 531	2 536	3 667	xx	3 850	xx
**TAPDUL**	Coformer not refined						
**TAPFAT**	Pamoic acid (NMP solvated)						
**TAPFEX**	Coformer not refined						
**TAPFIB**	C	xx	2 591	xx	3 411	xx	xx
**TAPFOH**	B	2 555	xx	3 350	xx	3 526	xx

**TAPFUN**	A	2 777	2 468	3 410	xx	3 835	xx
**TAPGAU**	B	2 520	xx	3 434	xx	3 554	xx
**TAPGEY**	A	2 549	xx	3 388	xx	3 874	xx
**TAPGIC**	A	2 743	2 527	xx	xx	xx	2 963
**WEMHII**	A	2 675	2 525	3 451	xx	3 626	3 513
**WEMHOO**	A	3 157	2 447	3 386	xx	3 458	xx
**WOSHIX**	B	2 625	xx			3 606	xx
**WOSHOD**	B	2 637	xx	xx	xx	3 626	xx
**WOSHUJ**	B	2 626	xx	xx	xx	3 655	xx
**WOSJAR**	B	2 620	xx	xx	xx	3 636	xx
**WOSJEV**	B	2 628	xx	xx	xx	3 640	xx
**WOSJIZ**	A	2 653	2 563	3 355	xx	3 498	xx
**WOSJIZ01**	A	2 664	2 534	3 474	xx	3 528	xx
**XEMYIY**	Pamoate ester						
**ZECMEC**	A	2 693	2 596	3 448	xx	3 585	xx
**ZECMIG**	A	2 698	2 604	3 438	xx	3 601	xx
**ZECMOM**	A	2 695	2 596	3 459	xx	3 566	xx
**ZECMUS**	A	2 679	2 611	3 428	xx	3 552	xx
**ZECNAZ**	A	2 682	2 594	3 460	xx	3 564	xx
**ZECNED**	A	2 691	2 599	3 438	xx	3 529	xx
**ZECNIH**	A	2 689	2 596	3 440	xx	3 551	xx
**ZECNON**	A	2 688	2 597	3 449	xx	3 599	xx
**ZECNUT**	A	2 693	2 596	3 435	xx	3 556	xx
**ZECPAB**	A	2 695	2 594	3 442	xx	3 563	xx

Table S17. Statistical parameters extracted from the CSD survey.

**HB - N**			**HB - O**			**π - π Hetero**			**π - π Homo**			**CH - π Hetero**		
**A**	**B**	**C**	**A**	**B**	**C**	**A**	**B**	**C**	**A**	**B**	**C**	**A**	**B**	**C**
80%	95%	50%	80%	5%	50%	92%	43%	50%	98%	10%	50%	88%	86%	50%
2.707	2.647	2.585	2.527	2.688	2.591	3.431	3.507	3.341	3.515	3.466	3.456	3.593	3.581	3.795
